# The Legacy and Future of Aluminum Alloys: Space Exploration and Extraterrestrial Settlement

**DOI:** 10.1021/acsmaterialsau.5c00139

**Published:** 2025-10-26

**Authors:** Matheus A. Tunes

**Affiliations:** Department Metallurgy, Chair of Nonferrous Metallurgy, 27268 Montanuniversität Leoben, Franz-Josef-Straße 18, 8700 Leoben, Austria

**Keywords:** aluminum alloys, space materials, metallurgy, materials science, extreme environments

## Abstract

The metallurgy of aluminum alloys has long shaped the history of space exploration. Their combination of low density, high strength, and excellent manufacturability and formability makes them indispensable for structural components in satellites and spacecraft. However, the next era of human space exploration, spanning long-duration deep-space missions and extraterrestrial settlement, poses unprecedented new challenges, including radiation damage and shielding, thermal cycling, micrometeoroid impacts, hydrogen embrittlement, and other degradation forces acting in synergy. Addressing these issues requires the reinvention of aluminum metallurgy, tailored to space environments. This perspective adopts a multidisciplinary approach, tracing the evolution of aluminum alloys in spaceflight, evaluating their performance under space-specific degradation mechanisms, and outlining future design strategies that integrate insights from chemistry, physics, metallurgy, and materials science to develop the next generation of space materials.

## Graphical abstract

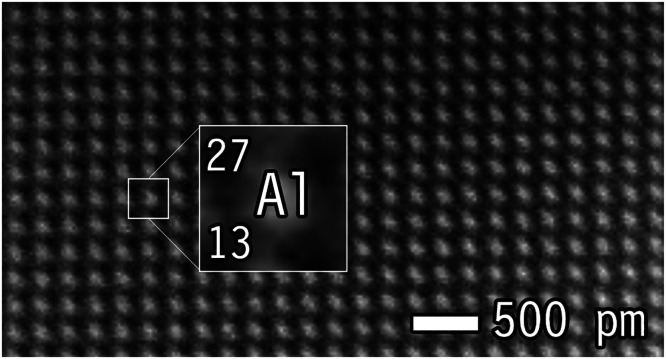

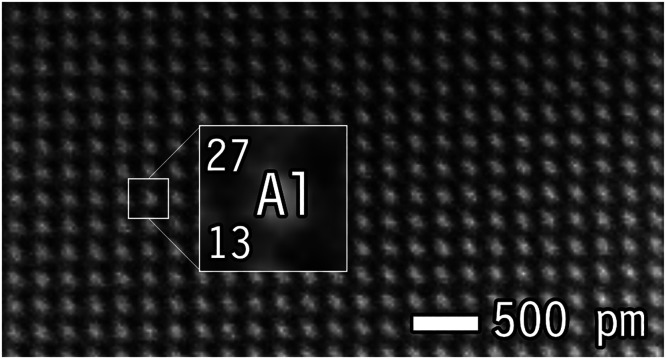

## Introduction

Humanity has long cultivated a strong fascination with venturing into space, and the realization of this ambition is highly dependent on advancements in the fields of metallurgy and materials science. Over the past decades, these disciplines have undergone profound development, consistently targeting the enhancement of material performance, focusing on greater mechanical resilience, economic efficiency, availability, recyclability, sustainability, and overall reliability. Historically, progress in these areas underpinned critical breakthroughs in early aerospace efforts, supporting innovations in flight dynamics, propulsion systems, and satellite and spacecraft design. Today, the renewed momentum of global space exploration initiatives—in part driven not only by state-sponsored programs, but also by the private sector—is rapidly advancing across several nations beyond the known spacefaring club, featuring nations such as the United States, China, India, and Russia.^2^

Rathnasabapathy et al.^3^ recently highlighted that a diverse group of developing nations across several regions of the world (including Latin America, Africa, the Middle East, and the Asia-Pacific) are becoming key players in the global space initiatives as well as in developing their own indigenous space-related industry. Countries including Argentina,^4^ Brazil,^5^ Mexico,^6^ South Africa,^7^ Egypt,^7^ Saudi Arabia,^8^ United Arab Emirates,^8,9^ Turkey,^10^ Israel,^11^ Australia,^12,13^ New Zealand,^14^ Indonesia,^15^ Thailand,^16^ Vietnam,^17^ and Malaysia^18^ are heavily investing in and expanding their domestic space initiatives, signaling a shift toward broader international engagement in space activities. In parallel, the United Nations plays a key role in promoting worldwide integration of nations toward the peaceful uses of space,^19–21^ but given recent geopolitical instabilities, many space-faring and developing space nations understand space technologies’ important role in modern warfare.^22^ Similarly to the first space race triggered in the Cold War by the technological innovation introduced by Soviet satellite Sputnik,^23^ Jenkins remarks that today’s space competition is primarily driven by the creation of new technologies, growing reliance on space assets, and rising commercial activity, with both governments and private actors seeking strategic and economic gains beyond mere prestige.^22^

A new space exploration and dominance race will inevitably introduce a fresh set of materials-related challenges. These include the need to develop systems capable of withstanding the extreme environmental conditions of interplanetary travel and extraterrestrial settlement. As a result, it is not too early to call that “space materials” has emerged as a distinct subdiscipline of both metallurgy and materials science with an increasingly vital field for research, development, and innovation.

The field of space materials has not arisen to address conventional challenges, such as mechanical strength or corrosion resistance. These issues are routinely tackled by the majority of metallurgists and materials scientists in designing materials for a wide variety of applications. Instead, the focus in space lies in developing advanced materials specifically engineered to withstand the extreme environmental conditions beyond Earth, understanding how these conditions may degrade their designed properties, and identifying strategies to mitigate such deterioration. Today, metallurgy and materials science offer an extensive portfolio of materials classified into categories such as metals, ceramics, polymers, and composites, many of which are either already used or actively being researched for numerous space applications. Historically, however, one subclass of metals has profoundly impacted and transformed space exploration: the aluminum alloys. One could even argue that space-related human activity would not have been possible without them.

In this paper, I am presenting a perspective on the past, present, and future of aluminum alloys and their role in future space exploration and extraterrestrial settlement missions. I want to share with you my enthusiasm and excitement about this marvelous field of research and human activity. First, I will summarize the key selection criteria that any material must exhibit for use in space, based on knowledge built upon the available literature on materials’ degradation in space environments. A noteworthy point deserves mention at this stage: literature and data on space materials remain limited and restricted, largely due to strict classification-of-information requirements—recently outlined as outdated and counterproductive barriers to addressing critical challenges in national space programs, exemplified by the challenges of the recently constituted U.S. Space Force.^24^ After addressing the materials selection criteria, I will present a concise historical overview of the use of aluminum and its alloys in space missions from the roots of the pioneering work of Robert H. Goddard^25^ to present times. The focus is on how aluminum alloys respond to the multiple degradation driving forces of the extreme environment of space. Finally, I will show the solved and unresolved challenges that aluminum alloys currently face related to future long-duration space missions, deep-space, and extraterrestrial settlement. I will conclude this perspective paper with a brief outlook on possible future research directions in the field of aluminum alloys for space applications, including some preliminary ideas developed and currently under investigation within our research group in Austria.[Fn fn1] It is worth noting that I will focus this review on the materials used as structural components of satellites, space probes, and spacecraft, rather than on materials for internal components or systems. Structural materials are the ones that will continue to enable human-based space exploration and future extraterrestrial settlement on other worlds.

## Primer in Materials Selection for Space Applications

Materials selection is a discipline of materials science that utilizes multidisciplinary concepts and strategies for designing and/or choosing the most appropriate (or optimal) material for a specific application. According to Professor Michael F. Ashby,^26^ approximately 2 × 10^5^ distinct materials had already been experimentally identified. If we consider the recently invented high-entropy alloys (HEAs), this number increases to the order of a “googolplex.”^27^ Narrowing down hundreds of thousands of known materials to those suitable for space missions represents an extraordinary scientific challenge. The space imposes stringent demands on materials, particularly due to the variety of degradation processes characteristic of the space environment. Most of the time, such a degradation process acts in synergy, which can further accelerate deleterious effects. The selection of materials for spacecraft and exploration systems, as well as the degradation processes found in space and their impact on material performance, have been thoroughly examined in recent reviews by Dever, Mouritz, Finckenor, Curreri, De Groh, and Ghidini.^28–33^ More recently, the outstanding challenges related to deploying metallic materials – especially aluminum alloys–in space applications were discussed by our team’s publications^34,35^ and academic works.^36,37^ In general, materials suitable for space missions must possess the following key characteristics.

### High Strength-to-Weight Ratio

The cost for any space mission is first averaged by the kilogram of material aboard the rockets at the time of launch. Jones reports that the pioneering Vanguard launch system from the United States achieved in late 1950s a payload cost of approximately 100 k$ kg^–1^ to reach Low-Earth Orbit (LEO).^38^ Since then, with the popularization of space exploration by both the third sector (space industry) and the people (space tourism), the price of payload has been on a sharp geometric decrease. SpaceX’s Falcon 9 has reportedly reduced the launch cost to LEO to approximately 3 k$ kg^–1^.^38^ Nevertheless, when costs per kilogram of material are a major material selection constraint, space program demand stronger-yet-lighter materials.

Figure 1A shows an Ashby map of strength versus density for mostly known classes of materials.^26^ The definition of strength varies across material classes, but it is commonly expressed as a measurable quantity derived from the material’s mechanical response. For metals, strength is often referred to as the yield strength, denoted by σ_*y*_, which can be defined as the stress at which a metal or alloy exhibits a permanent strain of 0.2%, marking the transition from elastic to plastic deformation. For ceramics and glasses, the modulus of rupture (MOR) is taken, while for polymers such as elastomers, the tensile tear strength is often referred to as strength. In this way, the Ashby map in Figure 1A provides a convenient way to compare the strengths of different material classes against their densities, both qualitatively and, to some extent, quantitatively.

**Figure 1:**
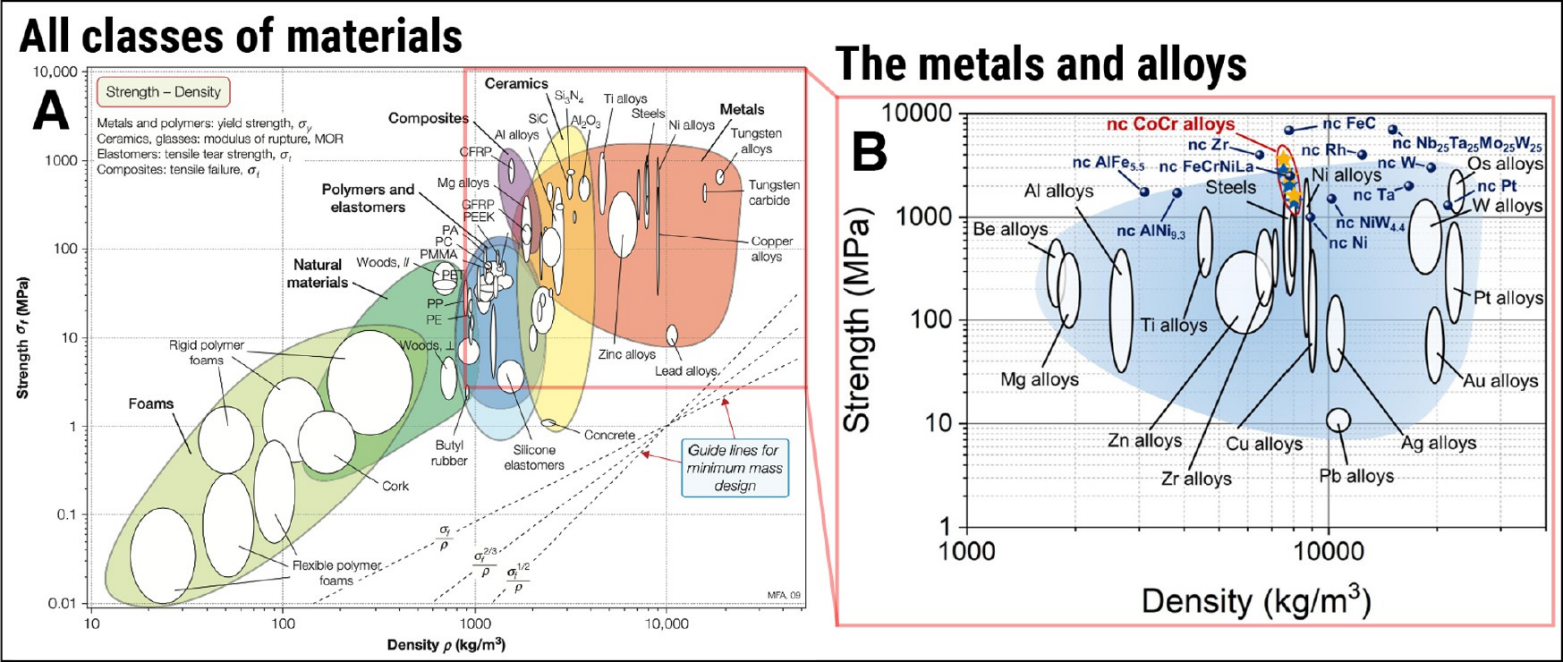
Lightweight yet stronger materials for space applications. (A) shows the Ashby map for strength versus density in (nearly) all classes of known materials.^26^ The Ashby map in (B)­shows the yield strength versus density for metals, alloys, and some select and new nanocrystalline (NC) alloys. Note 1: (A) was extracted and reproduced with permission from Ashby^26^ (Copyright 2021 Elsevier). Note 2: (B) was extracted and reproduced, modified from Wieczerzak et al.^39^ under an open access CC-BY-4.0 license (Copyright 2021 Wieczerzak et al.^39^).

Considering the application as structural materials in spacecrafts, probes, and satellites, upon analysis of the Ashby map in Figure 1A, one may conclude that ceramics, metals, and alloys feature higher strengths. Since most ceramics are inherently brittle, i.e., they cannot sustain significant plastic deformation without cracking, metals and alloys can offer the high strength required for use as structural materials in space applications. In fact, Wieczerzak et al.^39^ recently revised the metals and alloys section of this Ashby map, which I reproduce here in Figure 1B. Among the wide spectrum of high-strength metallic alloys, those with lower densities include beryllium, magnesium, and aluminum alloys. However, aluminum alloys are generally preferred for space applications due to a favorable combination of properties. Unlike beryllium, which is toxic,^40^ expensive,^41^ and difficult to process,^42^ aluminum offers excellent manufacturability, weldability, and cost-effectiveness.^43^ Compared to magnesium alloys—which are known to exhibit poor corrosion resistance^44^—aluminum can be tailored for higher corrosion resistance,^45,46^ present better mechanical stability at elevated temperatures,^47,48^ and currently feature a more mature and larger database of high-strength alloys aiming at aerospace applications.^47,49,50^ These advantages make aluminum alloys a widely adopted (and historical) choice for structural materials in spacecraft and satellites, considering their high strength-to-weight ratio, but this is only the first requirement a material must exhibit to be applied in space.

### Thermal Performance and Corrosion Protection

Structural materials for use in space must resist extreme temperature fluctuations while retaining their mechanical integrity. Adequate thermal properties should also encompass parameters such as specific heat capacity, thermal conductivity, behavior at cryogenic temperatures, and low coefficients of thermal expansion.^30,51–54^ Moreover, repeated exposure to thermal cycling can lead to thermal fatigue,^55^ which must be carefully considered during the design phase of the spacecraft. With surface temperatures plunging to cryogenic levels when in the Earth’s shadow and rising to approximately 150–180 °C under direct solar exposure, metallic alloys are subjected to wide temperature fluctuations while under LEO, and that may impact their microstructures, consequently their mechanical behavior.^56^ Comparable temperature ranges for metallic surfaces have been observed due to solar exposure at a distance of 1 A.U.[Fn fn2] from the Sun, outside the Van Allen belts.^57^ These extreme thermal cycles place stringent demands on aluminum alloys, which must retain microstructural stability by resisting phenomena such as recrystallization and/or grain coarsening, accelerated aging, and thermal fatigue.^34,35^

In order to protect crew members from extreme temperature fluctuations, coatings are also often applied onto aluminum alloys for thermal controlling.^52^ Black and white painting coatings were already designed and applied onto high-strength aluminum alloys in order to absorb (and dissipate) the energy from the Sun and/or increase the reflectance of the Sun’s thermal radiation. Although coatings enhance thermal performance, they also provide corrosion protection—another key degradation mechanism affecting space materials. Unfortunately, the problem of corrosion in space materials can begin as early as during exposure to Earth environments. Four of the most-used spaceports are shown in Figure 2A–D, depicting recent maps from Google Earth showing the Cape Canaveral (USA, Figure 2A), Alcântara (Brazil, Figure 2B), Baikonur (Kazakhstan, Figure 2C), and Shanxi (China, Figure 2D). In near maritime areas such as Cape Canaveral, Alcântara, and to a lesser extent in Shanxi, maritime saline corrosion emerges as a major degradation force of rocket and spacecraft materials. In fact, Calle^59^ emphasizes that Cape Canaveral is one of the most corrosive areas of the United States. The problem of corrosion of space materials while on Earth was already considered and addressed during the beginning of the space shuttle^60^ and solid-propellant rocket vehicles^61^ North American programs. The cosmodrome in Baikonur is located in a desert-steppe area of Kazakhstan, where dust erosion and associated corrosive processes are major concerns. Dust erosion may affect space materials even during flight.^62^ In this way, coatings and primer paintings are of paramount importance to enhance the resistance of aluminum alloys for space applications.

**Figure 2:**
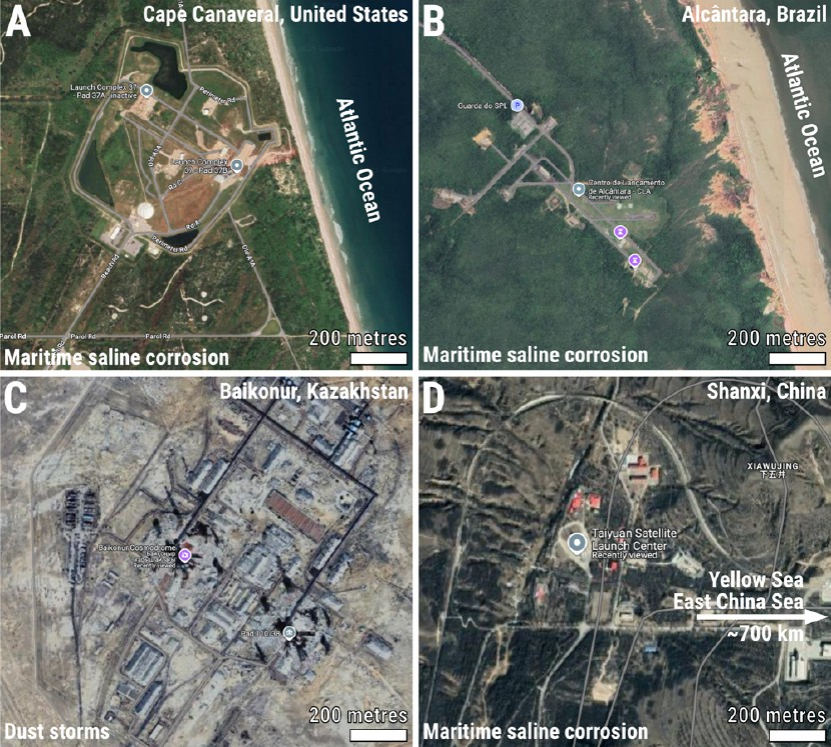
Terrestrial triggers of degradation in space materials. (A) Cape Canaveral (USA), (B) Alcântara (Brazil), (C) Baikonur (Kazakhstan), and (D) Shanxi (China) represent four of the most widely used and significant spaceports. (A, B, and D) are coastal or near-coastal sites (with the Taiyuan Satellite Launch Centre being the most inland), exposing materials to potential saline corrosion. In contrast, C Baikonur lies in a desert-steppe region, where wind and dust erosion are dominant degradation forces. Note: Locations identified using Google Maps (accessed July 2025) and reproduction permitted under the guidelines of fair use.^58^ Attributions for images/data are as follows: (A) Copyright 2025 Airbus/Maxar Technologies and Google; (B–D) Copyright 2025 Airbus/CNES/Maxar Technologies and Google.

Anodization strategies are often used to artificially grow protective layers in aerospace-grade Al–Cu alloys (namely the AA2024), which are naturally white,^63^ but it can be colored to black using immersion baths with cobalt acetate and ammonium hydrosulfide, followed by a sealing step with nickel acetate and boric acid.^53^ Figure 3A–H shows scanning electron microscopy (SEM) micrographs of a black-anodic coating on an AA2024 substrate. As studied by Goueffon et al., black coatings from anodization suffer from thermal strain after thermal cycling with temperatures in the range of ±140 °C,^53^ thus mimicking space conditions. These anodic layers are known to be highly porous at the nanoscale, making them susceptible to crack nucleation, propagation, and growth. Another area of research in materials science amid thermal fluctuations of spacecrafts and probes for deep space missions is metal–polymer interfaces. Byloff-Putz and co-workers^65^ are studying ways to enhance the robustness of aluminum-polyimide substrates via atomic layer deposition. Such coatings were already applied for thermal performance of the James Webb Space Telescope (JWST) sun shield and emerged as a potential area of further research in space materials, but the tribology with the metal substrate remains a major challenge.

**Figure 3:**
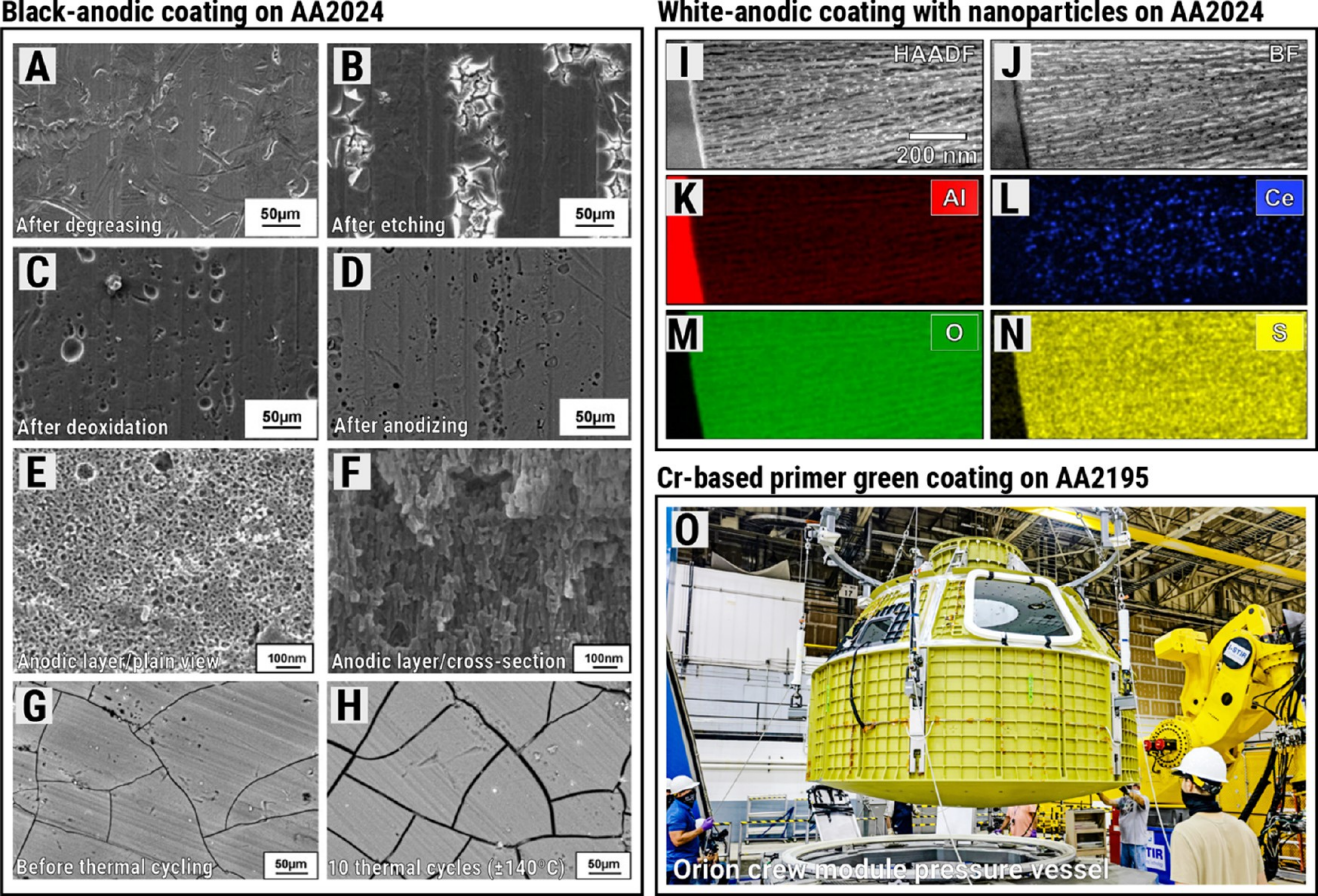
Coatings for thermal and corrosion protection of aluminum alloys. (A–H) SEM micrographs showing the steps to achieve a black-anodic protective layer on AA2024 (Al–Cu alloy) substrate: Goueffon et al.^53^ also tested the performance of this black coating under 10 thermal cycles varying the temperature within ±140 °C, and observed crack growth/widening caused by thermal stresses as noted in (G,H). (I–N) Shows STEM characterization of a strategy developed by our group to increase the corrosion resistance of white-anodic layer onto AA2024 substrate:^63^ The Ce nanoparticles shown in (L) within the anodic tubular pores are yet to be investigated as crack deflectors/inhibitors under thermal strain/cycling. The photograph in (O) shows NASA’s Orion crew module pressure vessel machined from AA2195 (Al–Li alloy) and protected with Cr-based green primer coating.^54^ Note: Micrographs (A–H) were extracted and modified with permission from Goueffon et al.^53^ (Copyright 2009 Elsevier), and micrographs (I–N) were extracted with permission from Prada-Ramirez et al.^63^ (Copyright 2020 Elsevier); Photograph in (O) is authored by NASA Johnson Space Center/Michael DeMocker and herein reproduced with courtesy of NASA under public licensing.^64^

In corrosion protection of aerospace-grade aluminum alloys, our group is investigating the influence of transition-metal nanoparticles embedded within the nanoporous tubules of white anodic layers synthesized on the AA2024 alloy, as illustrated in the scanning transmission electron microscopy (STEM) micrographs and energy-dispersive X-ray (EDX) maps in Figure 3I–N.^63^ Whether the fracture behavior of such nanoparticle-enhanced anodic layers presents potential to deflect or even stop crack propagation via nanophase engineering is pending further research. Another area of research to benefit the development of future space materials is special coatings exhibiting self-healing properties. Our research group has also recently shown that the corrosion resistance of the AA2024 alloy can be maximized with a top-protective sol–gel hybrid organic–inorganic coating combining Ce nanoparticles in the anodic tubular pores.^66^ Using scanning vibrating electrode technique (SVET) and local electrochemical impedance spectroscopy (LEIS), we have demonstrated that artificially introduced defects in the coating initiated a localized self-healing process, progressively restoring the original microstructure of the anodic layer over time after the damage.^66^

It is important to emphasize that recent efforts furthering anodization strategies on aerospace aluminum alloys emerge amid human health concerns. The photograph in Figure 3O shows the Orion crew module pressure vessel, which is made of an Al–Li alloy (AA2195), but it features a Cr-based green primer coating.^54^ Cr-based coatings are highly toxic for human health as they can cause severe cancer forms, which led them to be banned from the aerospace industry.^67^ NASA initiatives recently indicate a shift away from Cr-based coatings in space materials, as reported by Calle et al.^59^

### Hypervelocity Impact of Micrometeoroids and Space Debris

To endure the intense mechanical stresses encountered in space, such as collisions with micrometeoroids or orbital debris, candidate materials must combine excellent resistance to crack initiation with the ability to deform without fracturing under rapid loading conditions. Micrometeroids are of extraterrestrial origins and have an estimated velocity in the range from 4 to 51 km s^–1^ with an estimated average velocity reported to be around 20 km s^–1^.^33,68,69^ Conversely, orbital debris is another form of man-made junk arising as a result of mostly satellite breakups and spent solid rocket booster exhausts: they have an estimated average velocity of around 9 km s^–1^.^33^ Theories and models on micrometeoroid and hypervelocity impact on space materials were developed in the 1950s and 1960s at the peak of the Apollo program, with subsequent enhancements over the years with the development of the International Space Station (ISS). Bourton G. Cour-Palais dedicated his entire scientific career to such micrometeoroid impact problems.^70^ In the same way, theoretical modeling on the impact of space debris on space materials arose later in the 1980s, when the problem of space junk became noticeable, with the work of Kessler.^71,72^

Figure 4A shows the flux models for both micrometeoroid and space debris as a function of the particle diameter. For both models, the impact flux monotonically increases upon decreasing the particle size. Collisions between spacecraft surfaces and high-velocity micrometeoroids or debris typically release enough kinetic energy to not only vaporize the incoming object, but also generate a crater significantly larger (and often by a factor of 10 ^33^) than the original particle’s size. Mandeville *apud* De Groh et al. estimated that an aluminum fragment/debris moving at 6 km s^–1^ carries enough kinetic energy to induce vaporization and generate a crater approximately five times wider than the fragment itself. The impact flux is also a quantity for consideration here, as it expresses the rate of collisions per unit of time and area. Taking an impact flux of around 1 × 10^3^ impacts yr^–1^ m^–2^ for a micrometer-sized micrometeoroid, one can expect 2.74 impacts per day on a surface of 1 m^2^, which is quite a considerable danger for any space mission. The angular dependence of micrometeoroid and debris impacts also plays a key role in momentum transfer during collisions.^33^

**Figure 4:**
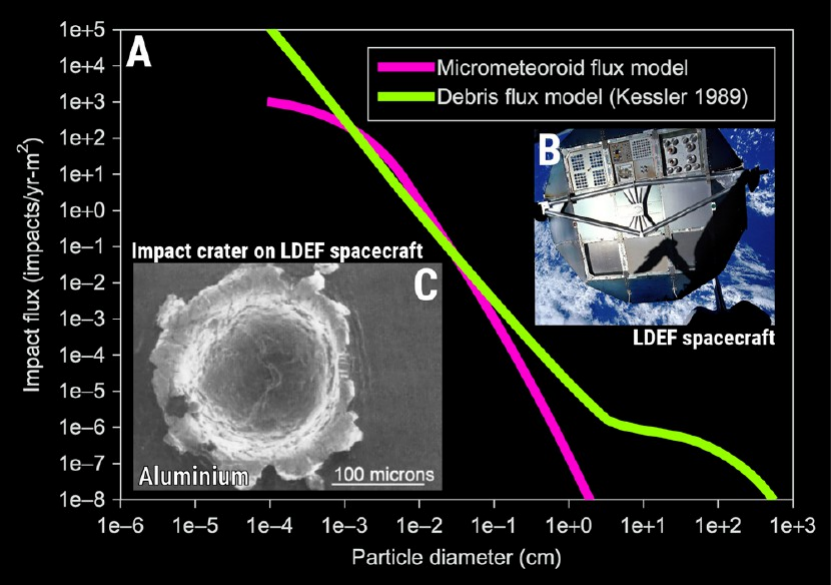
The danger of space junk and micrometeoroids. (A) Theoretical models on the impact flux of micrometeoroids^70^ and space debris.^71,72^ (B) The LDEF spacecraft.^73^ SEM image of an aluminum crater from hypervelocity micrometeoroid/debris impact aboard the LDEF spacecraft.^33^ Note: Plot in (A) and SEM micrograph in (C) were extracted and modified with permission from De Groh et al.^33^ (Copyright 2018 Elsevier). Photograph (B) is a courtesy of NASA’s public licensing.^73^

According to De Groh et al., impacts from micrometeoroids and space debris can pose a serious threat to mission integrity.^33^ If the localized mechanical damage caused by the impact of these micrometeoroids or space debris causes the rupture of power cables, electrical wires, or even the pressure vessel and/or fuselage of the spacecraft, this danger can also be considered life-threatening to future space explorers. The long duration exposure facility (LDEF)—Figure 4B^73^—was a spacecraft that exposed materials’ samples to the LEO conditions for 69 months. Figure 4C shows an SEM micrograph of an impact crater on aluminum resulting from hypervelocity particles. Damaged surfaces in aluminum are site-specific triggers for cracking and catastrophic failure. Literature on the mitigation of micrometeoroids and space debris impact on space materials is up-to-date, scarce, and incipient. De Groh et al.^33^ briefly reviewed these mitigation techniques in 2018. According to these authors, considering the possible rupture of power cables and wires, engineering redundancy has been applied as a mitigation strategy. Multilayer materials to form a “shield” to break up micrometeoroids and space debris apart is another strategy that was considered in the past.^74,75^ It is clear that this is an area of research of paramount importance to space materials, and it should receive significantly more attention in the near future.

### Material Supply, Fabrication, Processing, and Economic Considerations

A continuous and reliable supply of materials is essential to support long-duration space missions. Economic viability and production efficiency are key factors across the entire fabrication chain, encompassing procurement, shaping, machining, thermal treatments, quality control, and life-cycle management. Due to the restricted possibilities for in-orbit (or even deep space) repair of spacecraft structures and satellite components, selected materials must allow for straightforward replacement or exhibit inherent repairability.

In-Space Manufacturing (ISM) has gained attention over the past years, and several materials technologies are currently under intense development.^78–87^ Erik Kulu (factoriesinspace.com) subdivided ISM into three major areas:^76^ (i) lunch and/or resupply, (ii) on-orbit manufacturing, and (iii) use in-orbit and/or re-entry. In the first area, raw materials and consumables from Earth, space debris, asteroid mining, and also resources from the Moon are combined to manufacture new materials in space. Provided the availability of raw materials and resources—within orbital space stations and in future spacecrafts—future crew members may benefit from microgravity to aid in the manufacturing of new materials. The third area of ISM is a glimpse of the future applications: large-scale man-made space structures for the generation of power, food, alloys, and processing of materials back and forth from Earth, etc. Erik Kulu compiled a list of commercial enterprises engaged in or planning activities related to ISM and associated fields, as illustrated in Figure 5. This highlights the growing involvement of the private sector in the exploration and utilization of space resources.

**Figure 5:**
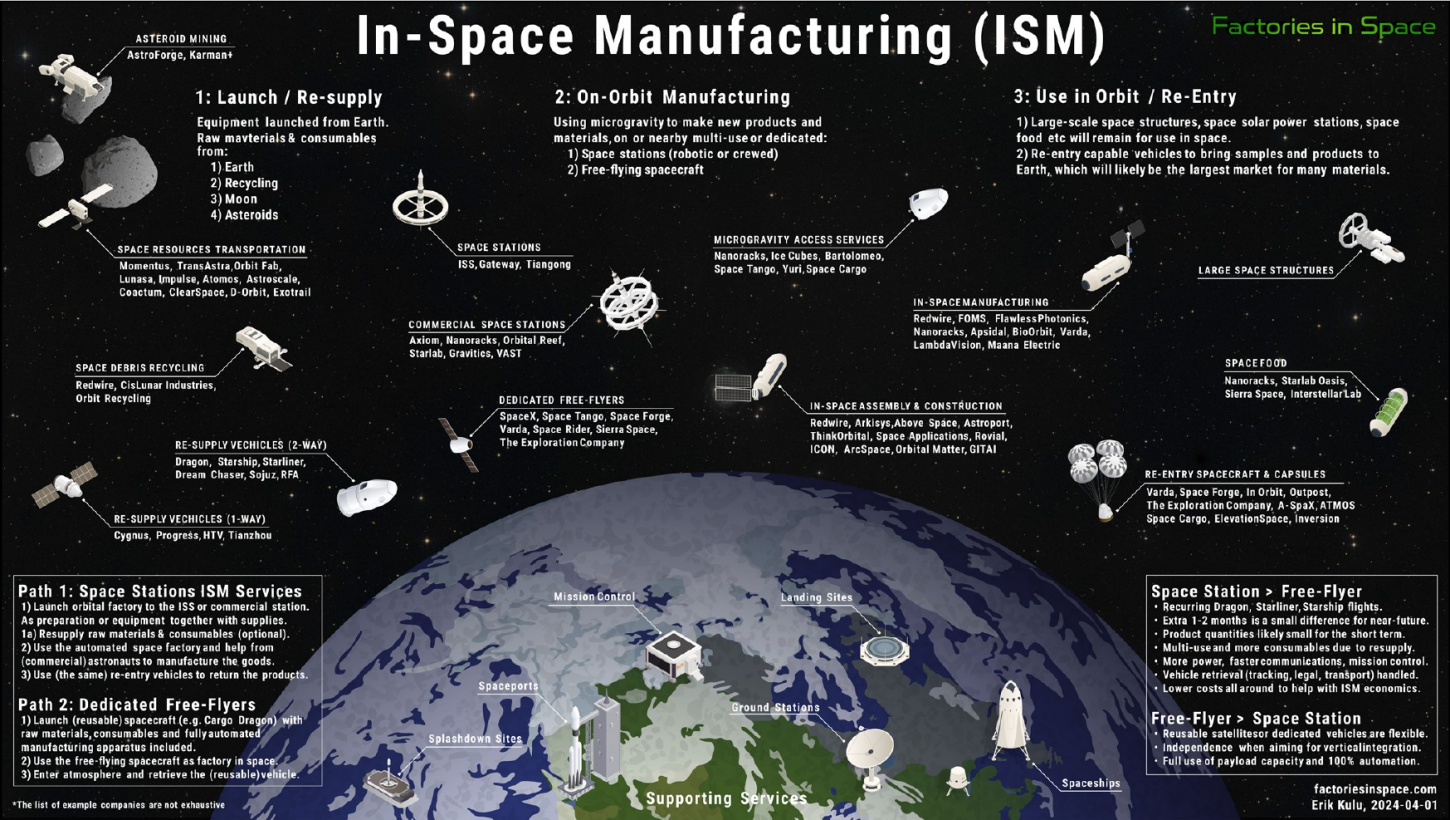
ISM offers real business opportunities. Stages of ISM and commercial enterprises engaged in and/or planning activities related to ISM. Note: Modified and reproduced with permission provided by the author: Erik Kulu (factoriesinspace.com).^76,77^

Additive manufacturing (AM) is an emerging science with high potential to revolutionize the manner in which humans carry out ISM activities. Four important review papers by Blakey-Milner et al.,^88^ Ishfaq et al.,^83^ Hoffmann et al.,^84^ and Subeshan et al.^86^ highlight the progress and development of AM in the space sector. They all emphasize the high applicability of AM for the fabrication of space materials, outlining its advantages of in situ/in loco production, geometric flexibility, and material efficiency. AM is recognized as a key enabler for future space exploration. While early in-space demonstrations have been limited to polymer-based processes, recent research has expanded toward processing metals and ceramics under reduced-gravity conditions.^86^ These efforts aim to overcome current technological gaps and enable sustainable fabrication and repair capabilities directly in space. In the aerospace sector, metal AM is already delivering substantial commercial and performance benefits, such as reduced cost and lead times, innovative lightweight designs, and enhanced component integration—supporting applications ranging from rocket engines to satellite components.^88^ Complementary to these industrial advances, research has shown that 3D-printed parts produced in space can match or even exceed certain ground-based properties, though challenges remain in process control, infrastructure, and materials behavior in microgravity. Collectively, these developments highlight both the progress and the outstanding challenges in establishing AM as a core technology for space missions. Materials intended for extraterrestrial settlements—such as those based on lunar regolith simulants—were also recently demonstrated to be produced using AM techniques.^85^

### Radiation Damage and Radiation Shielding in Space

Materials deployed in outer space are frequently exposed to intense radiation fields, including high-energy charged particles such as protons (*p*), electrons (*e*), alpha particles (α), and heavier ions alongside ionizing electromagnetic radiation like γ rays, X-rays, and infrared and ultraviolet radiation. This irradiation can be both from solar and interstellar origins. Effective protection against radiation must comply with the ALARA principle (As Low As Reasonably Achievable),^89^ which demands careful optimization of shielding properties considering the strict engineering requirements of spacecrafts as well as the need to protect human crew from biological effects of radiation. Consequently, the development of materials for space applications must balance efficient radiation shielding attenuation with the ability to endure structural and functional degradation caused by prolonged radiation exposure.

The Sun’s activity and behavior are continuously monitored by numerous international solar missions;^90^ however, the total number of past, ongoing, and planned missions dedicated specifically to this topic remains relatively limited. This is particularly noticeable given the vast scale of the solar system, the anisotropic and nonlinear nature of solar phenomena, the complexity of the near-Earth space environment, and the recent increase in space exploration by the third sector. Interestingly, this endeavor of tracking the Sun’s activity inaugurated an entire new field of research in Astronomy & Astrophysics known as “space weather.” In an analogy to the science of meteorology on Earth, space weather was defined by the U.S. Office of the Federal Coordinator for Meteorological Services and Supporting Research^91^ (*apud* Moldwin^90^):

*“*Space Weather refers to the conditions on the Sun and in the solar wind, magnetosphere, ionosphere, and thermosphere that can influence the performance and reliability of space-borne and ground-based technological systems and can endanger human life or health. Adverse conditions in the space environment can cause disruption of satellite operations, communications, navigation, and electric power distribution grids, leading to a variety of socioeconomic losses” ^90,91^

From both metallurgy and materials science perspectives, space weather demands new materials solutions for spacecraft and extraterrestrial habitats that will be continuously exposed to various forms of particle and electromagnetic radiation. Consequently, new material strategies that both mitigate radiation-induced microstructural damage and provide effective shielding are essential for space operations—particularly in the context of human missions.^30,32,34^

Although the literature on space weather is somewhat extensive, experimental studies specifically investigating the type, energy, and spatiotemporal flux of solar energetic particles (SEPs) have only become a significant focus in the past decades. The ejection and acceleration of particles by the Sun were first proposed theoretically by Biermann in 1951 ^92^ and later by Parker in 1958,^93^ based on analyses of cometary tails observed in orbit around the Sun. Experimental confirmation that the Sun indeed emits SEPs was independently performed by both the Soviet Union (Lunik 2 and 3) and the United States (Explorer 10 and Mariner 2).^94^ Alongside SEPs, galactic cosmic rays (GCRs) originate from sources beyond the solar system and consist of particles with higher kinetic energy than SEPs, along with some different species such as heavier ions,^95^ further compounding the radiation hazards posed by the solar wind. For missions within LEO, Earth provides natural protection against SEPs, GCRs, and electromagnetic radiation through its relatively uniform magnetic field, which acts as a natural shield via the Van Allen (VA) belts. These belts trap incoming radiation, diverge its path, and attenuate its flux, thereby reducing—though not eliminating—the risks such radiation poses to both materials and human health. Beyond the Van Allen belts, deep space missions can be fully exposed to the intense effects of SEPs and GCRs, necessitating more stringent material selection criteria to ensure adequate protection for both crew members and electronic systems. Moreover, unpredictable events like solar flares (SFs) and coronal mass ejections (CMEs) may significantly increase both the energy and flux of SEPs, posing an added risk to the forecast of space missions.^96^

An attempt to fully quantify the spectra of energy and flux of all SEPs and GCRs at a specific time and location in space is an inherently difficult task due to the variability and complexity of this phenomenon. In this work, I do not aim to define the exact particle types, energies, or fluxes that may be encountered at specific locations in space. A good overview of the danger of radiation within the solar system is from the knowledge collected from previous and current solar missions. Great compilations of space weather data can be found in specialized journals,^92,93,95,99,102–124^ books,^90^ and handbooks.^98^ These measurements have been performed in multiple space locations, such as in LEO,^98^ around the Moon with CRaTER^96^ (Cosmic Ray Telescope for the Effects of Radiation), and recently closer to the Sun with the Parker Solar Probe (PSP).^124^ Nevertheless, the mature field of space weather offers a solid foundation for the radiation environment of the solar system, and such information can be used for informed materials design, which is the primary aim of this work. Table 1 contains an overview of the radiation environment in the solar system’s radiation spectra from both solar and interstellar origins compiled from data in the literature.^97–102^

**Table 1:** An Overview of the Radiation Environment of the Solar System

**source/occurrence**	**particle species**	**observed energy range**	**observed flux range**	**refs**
sun/solar energetic particles^a^	*p*, *e*, α	0.005–1 MeV (solar wind)	10^–3^–10^12^ cm^–2^ s^–1^	^97–101^
		≤1 GeV (SFs and CMEs)		
sun/trapped radiation (LEO)^b^	*p*, *e*	0.5–100 MeV	10^–1^–10^8^ cm^–2^ s^–1^	^97,98,101^
interstellar/galactic cosmic rays	*p*, α, heavier ions	GeV to TeV	10^–10^–10^0^ cm^–2^ s^–1^	^98,102^

a Trace heavy ions (C, N, O, Ne, etc.). Sharp flux and energy increase during SFs and CMEs.

b Flux and energy change with altitude.

Figure 6A shows the energy spectrum and flux of solar wind protons at 1 A.U. as tabulated by Sznajder^100^ using data from four different space missions dedicated to the solar weather phenomenon: i.e., SOHO,^125^ ACE,^126^ WIND,^127^ and DSCOVR.^128^ Sznajder^100^ reports a 240% variation in proton flux magnitude across data from such missions, underscoring the challenges in accurately estimating the Sun’s activity related to SEP emissions, and he also discusses the implications of these uncertainties in planning future deep space missions. An extra layer of danger is added when SFs and CMEs are taken into consideration. Figure 6B shows what happens with both the energy and flux of solar protons during an SF. These measurements reported by Schwadron et al.^96^ were taken by CRaTER around the Moon, and they report on solar proton energies as high as 0.5 GeV with a 4-fold magnitude increase in proton fluxes. CRaTER also uses this data to estimate the equivalent dose of radiation in 1 g cm^–2^ of H_2_O as a proxy to comparable doses in both the eye lens and the skin, and 10 g cm^–2^ of H_2_O as a proxy to blood-forming organ doses.^96^ As shown in the first plot in Figure 6B, the radiation dose rate can spike to 10^2^ cGy day^–1^ during an SF event. In 1 day, this would lead to a dose of 1 Gy in both the eye lens and skin. The medical literature reports that an adult can tolerate 5 Gy of dose in the eye lens^129^ and around 15 Gy in the skin^130^ without any deterministic biological effects of radiation.

**Figure 6:**
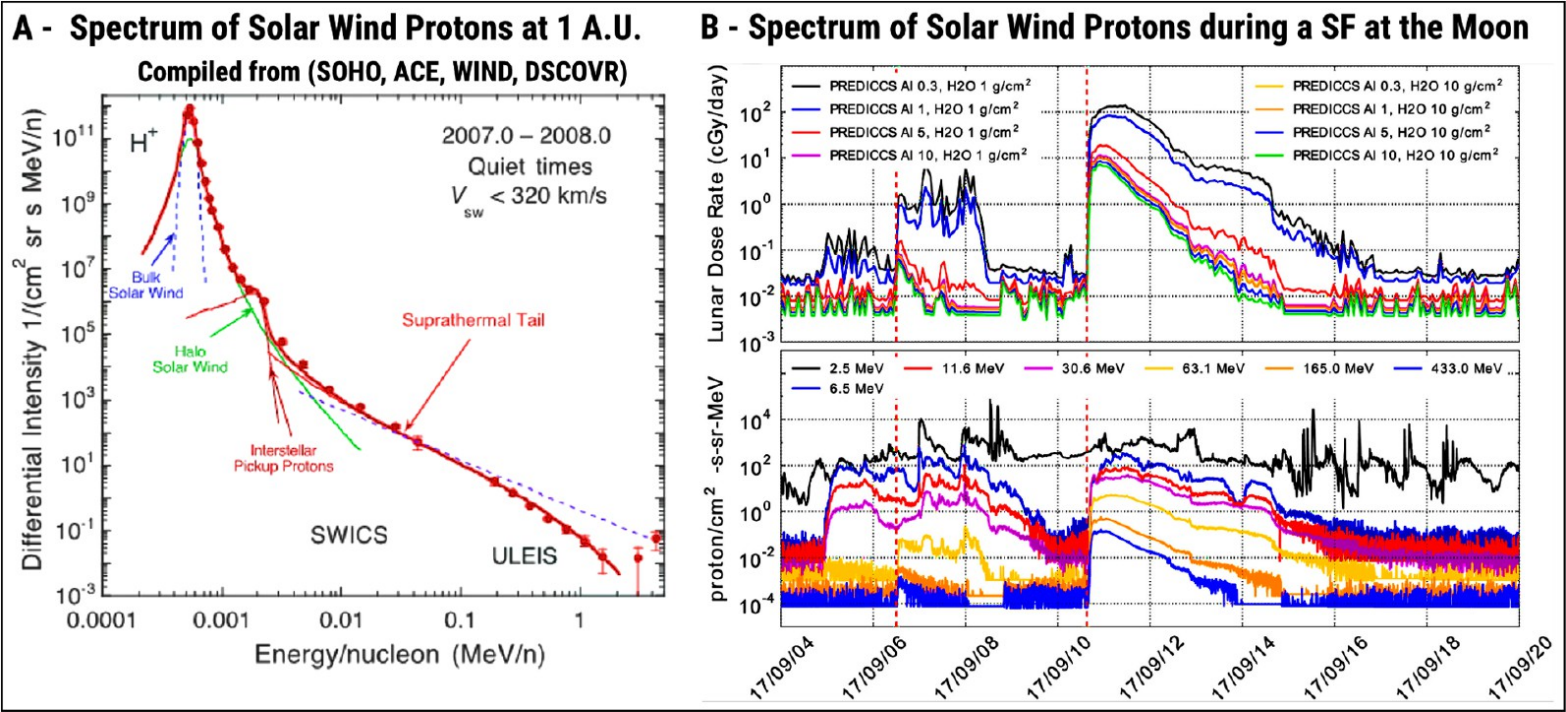
The radiation environment of the solar system. Under normal conditions, the Sun constantly emits solar wind protons throughout the solar system, and the energy and intensity spectrum of solar wind protons is shown in (A). Under abnormal conditions, the Sun experiences SFs and CMEs, leading to an increase in both energy and flux of solar protons (B) for a certain period of time, consequently increasing the radiation dose rate for both materials and human crew, adding an extra layer of danger for future space missions. Note: (A) and (B) were extracted and reproduced with permission from Sznajder^100^ (Copyright 2023 Elsevier) and Schwadron et al.^96^ (Copyright 2018 Elsevier).

The particle irradiation environment of the Moon is worsened during SFs, as shown by Schwadron et al.^96^ Human crew members could be subjected to 20% of the eye lens dose limit in only 1 day if proper radiation shielding measurements are not applied. Figure 7 illustrates this important property of aluminum as a space material, which is to provide shielding for future spacecraft and human crews. Based on the 1-month accumulated dose around the Moon,^96^ CRaTER data estimate that the aluminum thickness must be doubled to reduce the dose to the eye lens and skin to approximately half of the 30-day occupational limits. Such considerations of radiation dose and shielding are of paramount importance for future human-based missions to the planet Mars.^117^ All these facts suggest that the requirement for aluminum plates to absorb radiation introduces a secondary challenge in space materials research: radiation damage within the aluminum microstructure. The resulting microstructural defects may degrade the shielding over time and potentially lead to failure during a mission and put human crews in danger of high levels of radiation exposure. This issue highlights a critical selection criterion for space materials, the dual requirement of providing effective shielding while simultaneously resisting microstructural degradation caused by radiation exposure.

**Figure 7:**
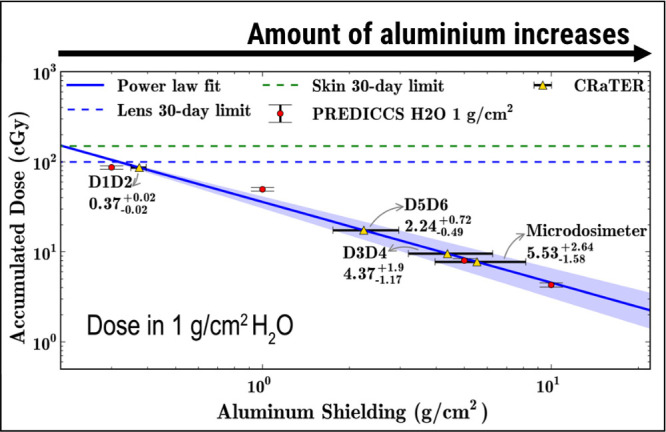
Aluminum as a space material for radiation shielding. Accumulated radiation dose in September 2017 was measured at the Moon by the CRaTER, where the effects of SFs were detected and quantified. Considering the 1 g cm^–2^ of H_2_O as a model for the eye lens and skin, the amount of aluminum for shielding SEP should more than double to decrease such a dose to half of the 30-day limit. The solid blue line is a power law fit, while the discrete points are CRaTER measurements. Note: The plot was extracted, modified, and reproduced from Schwadron et al.^96^ (Copyright 2018 Elsevier).

## Aluminum Alloys in the Extreme Environment of Space

In the previous section, I outlined the key criteria that candidate space materials must fulfill. I examined these in the context of aluminum and its alloys, which are already established as space materials due to their proven performance. I now turn to a brief historical overview of aluminum alloys in space applications, followed by recent developments from our group aimed at supporting metallurgy and materials research, the future deep-space, long-duration missions, both for outer space exploration and human settlement on extraterrestrial worlds. This section focuses on the response of aluminum alloys to energetic particle irradiation, which is arguably one of the most demanding challenges a space material must withstand. As an important radiation shielding material in space activities, a great part of the kinetic energy that aluminum absorbs from SEPs is converted into radiation damage. Therefore, the greatest challenge of future human-based space exploration is to develop an aluminum alloy that is capable of shielding SEPs and—at the same time—exhibits strong radiation tolerance.

### Extraterrestrial Origins of Aluminum

The chemical element aluminum has only one primordial isotope, ^27^Al, and as for all elements of the periodic table, it is of extraterrestrial origins. The star nucleosynthesis of ^27^Al takes place in the inner core of massive stars (≈ 8–10M_⊙_) where elements such as ^26^ Mg may absorb protons, thus producing ^27^Al via nuclear fusion reactions. Baumüller and Gehren argued that the synthesis of ^27^Al requires either (i) an explosive event or (ii) neutrons within a star that undergoes stationary burning processes.^131^ Interestingly, aluminum ranks as the twelfth most common element in the universe and is the third most prevalent among those with odd atomic numbers, following hydrogen and nitrogen. The primordial isotope ^27^Al is the 18th most abundant atomic nucleus in the cosmos.^132^ Explosive events such as supernovae launch element-rich asteroids into space. Chondritic is a class of meteorites containing aluminum-rich inclusions.^133^ By weight, aluminum is the most abundant metal in Earth’s crust.^134^

### From Scientific Discovery to Industry and Science Fiction

The chemical reaction of aluminum chloride with potassium amalgam (eq 1) was carried out by the Danish chemist Hans Christian Ørsted,^135^ yielding the discovery of the chemical element aluminum in 1825.^136^ Two years later, the German chemist Friedrich Wöhler^137^ reported on his own difficulties in attempting to reproduce the experiments from Ørsted.^138^ In his own skeptical words:

“I repeated Ørsted’s experiment, but did not obtain a clear result. When potassium amalgam was distilled after it had been heated with aluminum chloride, a large molten metal mass remained. However, when the heat was increased to higher temperatures until glowing, it evaporated into green vapors and distilled over as pure potassium. Therefore, I looked for another method, but without wanting to say, it is not possible to reduce aluminum with this method” – Friedrich Wöhler (1827)^138^.

Years after Ørsted’s discovery and attempted validation, it was only in 1856 that the French chemist Henri Sainte-Claire Deville^139^ devised a new method for the industrial production of aluminum,^140^ which involved the use of sodium (instead of potassium) for the reduction of aluminum chloride (eq 2): a method proved more efficient and cheaper than Ørsted’s, but yielding aluminum with lower purity levels. Industrial-large-scale aluminum production began in 1886, when Paul Héroult and Charles Martin Hall independently developed the electrolytic Hall–Héroult process for directly reducing alumina (aluminum oxide) to metal.^141^ In 1889, Carl Joseph Bayer finally introduced a method to extract pure alumina from bauxite, which is the natural mineral containing aluminum, thus scaling up the industrial production of aluminum. Today, although a significant share of industrial aluminum production comes from recycled scrap, primary aluminum is still produced using a combination of the Bayer and Hall–Héroult processes.^142^

$${\mathrm{AlCl}}_{3}+3K\to \mathrm{Al}+3\mathrm{KCl}$$ 1

$${\mathrm{AlCl}}_{3}+3\mathrm{Na}\to \mathrm{Al}+3\mathrm{NaCl}$$ 2

Following the discovery and industrial production of aluminum, interestingly, the first to propose its use in space was not a scientist but a writer: Jules Verne.^143^ Less than 10 years after Deville’s industrial production and popularization of aluminum, in the 1865 Verne’s book “From Earth to the Moon,”^144^ in the middle of a discussion within the “Baltimore Gun Club”, the characters of the story were arguing about selecting the best material to be used as a space projectile, to be launched into space toward the moon. After opposition to the use of cooper (they scientifically knew space materials had to be lightweight), the character Barbicane suggested aluminum as

“it is easily wrought, is very widely distributed, forming the base of most of the rocks, is three times lighter than iron, and seems to have been created for the express purpose of furnishing us with the material for our [space] projectile” – Jules Verne (1865)^144^.

### Precursors of Light-Weighting for Space Applications

From scientific fiction to reality, Robert H. Goddard was the first pioneer in space research in the early 1900s. Working on his farms in New Mexico and Massachusetts, he was able to launch the basis of modern rocketry without any dedicated U.S. Government funding, but only with his own curiosity, passion, and with the support of his team, and important financial aid from both the Guggenheim and Smithsonian foundations, he revolutionized the history of aerospace engineering. His list of achievements goes well beyond inventing modern rocketry. In his personal papers—available to consult thanks to the Smithsonian foundation^145^—Goddard details his ingenious invention (and secret) to allow liquid-propellant rockets to fly: rocket weight reduction using thin-walled fuel tanks made of aluminum alloy wound with high-tensile-strength steel wires. More than 100 years after Goddard, aluminum-lithium alloys are the material of choice for rocket fuel tanks. Additions of lithium to compose an alloy reduce the density of pure aluminum with concomitant increase in hardness and stiffness^146^ via three effects: solid-solution strengthening, strain hardening, and precipitation.^147,148^

In the early 1930s, Werner von Braun and Hermann Oberth's efforts were to militarize rocketry and make new weapons for the Third Reich. Their ballistic missile V-2 was the first man-made object to reach outer space. Working in Peenemünde under direct funding of Nazi Germany, Braun and Oberth faced several challenges regarding the resilience and durability of the aluminum alloys (and also steels) used in their rockets. Their team innovated in anodizing solutions to increase the corrosion resistance of aluminum alloys at high temperatures and when in contact with liquid propellants. In the early 1940s, with the start of the war, their team suffered from limited access to raw materials, thus hindering the progress of the V-series ballistic missiles.^149^ With the end of World War II, the Peenemünde’s team emigrated to USA via Operation Paperclip,^150^ where their led further military rocketry research in the U.S. Army Ballistic Missile Agency (Hunstville, Alabama).^151^ Werner von Braun’s leadership as first director of the NASA’s Marshall Space Flight Center were of paramount importance to the Apollo program and the first human landing on the Moon. After the war, space materials research entered the Cold War era.

### Aluminum in Cold War’s Space Race

Although the United States succeeded in landing the first humans on the Moon in 1969, the Soviet Union achieved many of the first milestones in space exploration, including the first satellite, the first mammal in space, the first human in space, the first woman in space, the first spacewalk, and many others, underscoring its paramount role in the early stages of the space race. One of the major secrets behind the Soviet success in space was the focus on the development of its national metallurgical industry, especially the industry of aluminum and its alloys. During the space race (1957–1969), it is not an overstatement to say that metallurgy played a pivotal role in safeguarding state secrets. The nations with the “best alloys” were able to leapfrog the main innovations. In 2014, the Central Intelligence Agency (CIA) released a previously classified handbook dated from August 1959, which provides a comprehensive compilation of soviet metallic alloys’ definition and classification—perhaps the best scientific handbook[Fn fn3] of metallurgy and alloy compositions in the mid-20th century—from the era when the soviets and North Americans were beginning to explore space.^152^

The two major scientific and technological innovations enabled by aluminum and its alloys in space were Sputnik-1, the first artificial satellite, and Vostok-1, the first manned spacecraft, both shown in Figure 8.^153,154^ The body of the Sputnik-1 was composed of two hemispherical shells of 58 cm diameter made from the AlMg6 ^155^ alloy, which was developed by the soviet metallurgist Joseph Naumovich Fridlyander^156^ and it combined good formability, and both high ductility and specific strength.^155^ Five years later, the spacecraft Vostok-1 harbored Yuri Alekseevich Gagarin to complete the first space flight in LEO. The Vostok-1 is a combination of eight different aluminum alloys. The structures of the descent module and the instrumentation bay used the AMg6 alloy. Pipeline systems incorporated AMg2 and AMg3 grades, while D16, D19, AK6, and AK8 alloys were selected for structural components such as the power set and transport system. In crewed spacecraft, components like impellers within the life support systems were produced from aluminum alloy B124.^155^

**Figure 8:**
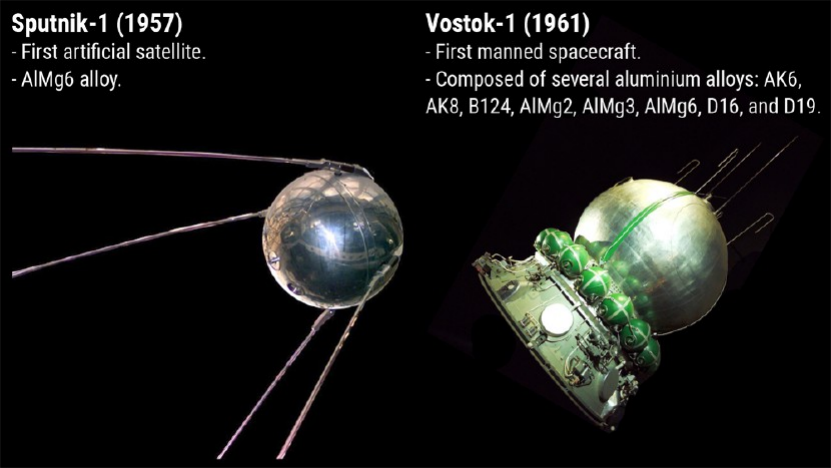
Under aluminum’s shield, humanity conquered space. The chemical element aluminum and its alloys gave mankind the lightness and the strength needed to conquer space. The Soviet Union’s Sputnik-1 was made of AlMg6 alloy with good formability, weldability, and corrosion resistance. Upon developing the metallurgy of aluminum, the Soviets launched the Vostok-1 five years later, the *magnum opus* of human engineering, composed of at least eight different (both cast and wrought) aluminum alloys. This spacecraft allowed Cosmonaut Yuri Gagarin to travel in LEO for the first time in human history. Note: The photo of the Sputnik-1 satellite is a courtesy from Pixabay^153^ (free for use). The photo of the Vostok-1 is a courtesy of Wikimedia/Pline^154^ (copyleft, multilicense with GFDL, and CC-BY-SA-2.5).

The composition of some of these alloys is shown in Table 2.^152^ Comparing them with the west-equivalent alloys, one can see that these can be classified as wrought age-hardenable aluminum alloys. The AA2xxx and AA7xxx series alloys are now conventional aerospace-grade materials for high-strength applications, whereas the AA5xxx series is known for good formability yet lower strain levels. While the AA2xxx and AA7xxx are heat-treatable alloys, the AA5xxx series is non-heat-treatable.

**Table 2:** Chemical Composition^a^ of Soviet Aluminum Alloys and Western-Equivalent Classification^152^

**alloy**	**Al**	**Cu**	**Fe**	**Si**	**Mn**	**Mg**	**Zn**	**Ni**	**others**	**west-equivalent alloy**
AK6	balance	1.8–2.6	0.7	0.7–1.2	0.4–0.8	0.4–0.8	0.3	0.1		
AK8	balance	3.9–4.8	0.7	0.6–1.2	0.4–1.0	0.4–0.8	0.3	0.1		AA2014
D16	balance	3.8–4.9	0.8	0.8	0.3–0.9	1.2–1.8	0.3	0.1	Fe+Ni at 0.5	AA2024
AMg2/3/6	balance	0.1	0.4	0.4	0.15–0.35^b^	2.0–6.5	0.2		V 0.02–0.2	AA5xxx
B95^c^	balance	1.4–2.0	0.5	0.5	0.2–0.6	1.8–2.5	5.0–7.0		Cr 0.1–0.25	AA7075

a Maximum percent unless given a range.

b Mn or Cr.

c I have not found the composition of Vostok-1’s alloy B124. Arguably, the B95 is the closest one in the class with open source data available.

On the American side of the space race, great success of the U.S. Space Shuttle Orbiter (SSO) program can be attributed to aluminum’s metallurgy, as can be seen in Figure 9.^157^ The SSO project comprised different aluminum alloys and it has been stated by Schneider and Miller^158^ as essentially a spacecraft made out of conventional aluminum (and alloy) sheets, graphite epoxy (for both the cargo bay doors and the orbital maneuvering system pods) and titanium (for the thrust structure). What is even more fascinating about SSO is that new shapes were invented to support structural applications with concomitant reduction in weight. These were machined aluminum webs and honeycombs. Apart from the aluminum alloy classes also used by the soviets, the SSO featured parts made of AA6061 (Al–Mg–Si) in peak strength condition—the T6 temper—to compose the orbiter radiator system.^159^

**Figure 9:**
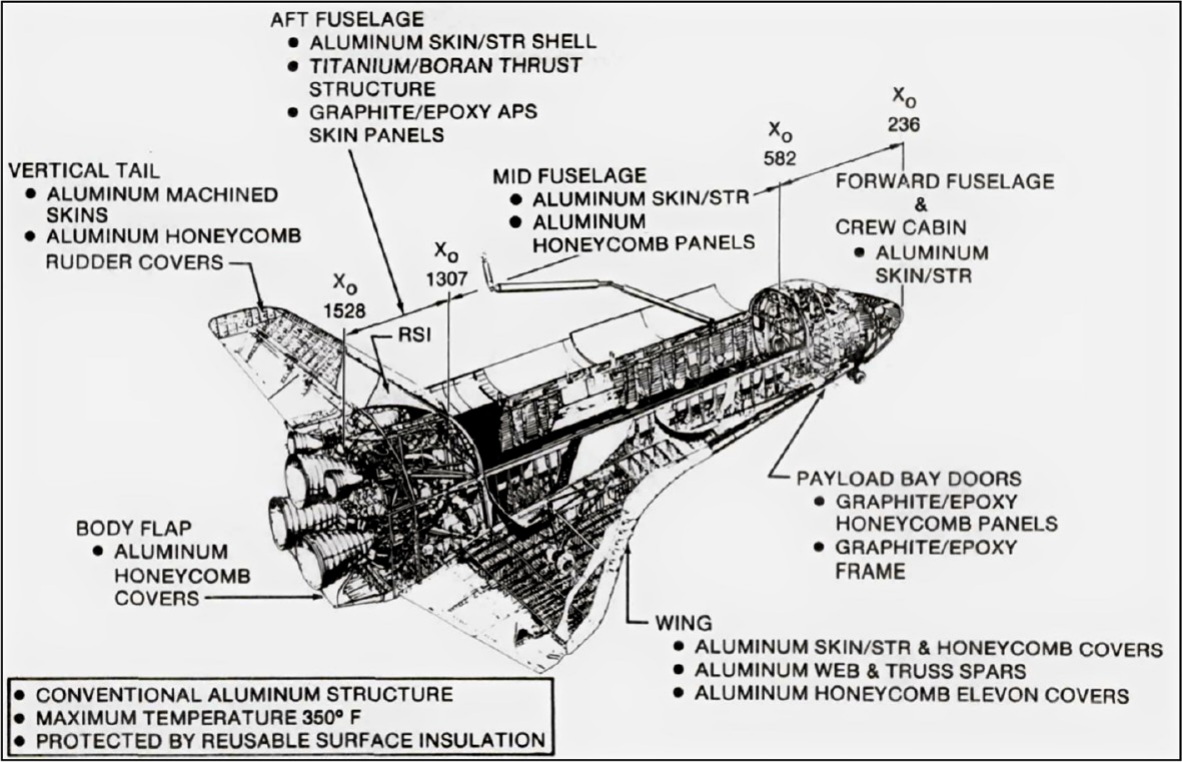
Aluminum and the space shuttle. The engineering project of the SSO was essentially composed of aluminum and its alloys as structural materials, graphite epoxy, and titanium. Note: Reproduced from work performed by the U.S. Government and Glynn et al.,^157^ public use permitted.

Given the past success both the Soviets and North Americans achieved in short-term space missions using aluminum alloys, a question remains: would these alloys survive the high radiation damage and shielding levels expected for long-term future space missions aimed at deep-space exploration and extraterrestrial settlement?

### Brief Review on the Irradiation Resistance of Commercial Aluminum Alloys

Considering the past literature on the effects of irradiation on commercial aluminum alloys, a limited number of papers report exclusively on the effects of neutron irradiation on some select alloy classes, such as AA2xxx, AA5xxx, AA6xxx, AA7xxx, and also on high-purity aluminum^160–168^ to be used in low-temperature reactors such as materials test reactors (MTRs). Kolluri recently wrote a review paper specific to the effects of neutron irradiation on AA5xxx and AA6xxx alloys.^168^ As covered in the previous section, the major driving force for radiation damage in space is from solar wind protons, electrons, some heavier ions, and GCRs. SFs and CMEs pose aggravations to fluxes and energy levels. Considering the use of aluminum alloy in space, neutron irradiation is only a secondary problem, and I will come back to this topic later on.

Thermodynamic stability plays a critical role in determining how materials respond to irradiation. Under energetic particle bombardment, the continuous ballistically induced generation of vacancies and interstitials drives diffusion far beyond thermal equilibrium, accelerating phase transformations, segregation, and precipitation. If a microstructure is thermodynamically metastable, irradiation tends to promote decomposition into energetically more favorable states,^169^ often accompanied by microchemical inhomogeneities and the nucleation of secondary phases. Such transformations can alter the mechanical properties, increase embrittlement, and compromise the long-term structural integrity. Conversely, materials with thermodynamically stable phases are less prone to irradiation-induced decomposition as the system lacks a strong driving force for phase separation even in the presence of high defect concentrations. Thus, designing alloys with stable microstructures is essential to mitigate irradiation damage and ensure performance in extreme environments.

Nevertheless, the knowledge accumulated^160–168^ on neutron irradiation of commercial aluminum alloys allows us to grasp what type of radiation effects these alloys typically experience in an energetic particle irradiation environment. Particle irradiation affects age-hardenable aluminum alloys through a variety of microstructural changes. First, irradiation-generated point defects, such as vacancies and interstitials, can interact and bind with solute atoms, delaying the usual segregation and precipitation processes. Second, neutron activation and transmutation reactions (e.g., see eq 3) accelerate the formation of Guinier–Preston zones and other strengthening phases like Mg_2_Si (the β-phase) or Al_2_Cu (the θ-phase), particularly in Al–Mg and Al–Cu-based systems, which may lead to embrittlement due to excessive precipitation. Third, radiation damage—manifesting as dislocation loops, voids, and other defects—serves as a favorable site for heterogeneous nucleation of precipitates, as atomic mobility in aluminum remains high even at low temperatures. Lastly, irradiation may alter or dissolve existing hardening phases due to thermal spikes and defect cascades, sometimes causing unexpected softening, particularly in alloys with initially higher strength/hardening levels.

Al27(n,γ)Al28→Si28+β− 3

Studies on the radiation response of commercial aluminum alloys to low, medium, and high energy protons, thereby directly mimicking solar wind protons, are scarce. The seminal work of Lohmann–Sommer et al.,^170^ published in 1986, continues to be one of the most influential works in the field. These authors irradiated both AA6061-T6 and the AlMg3 alloys with 600–800 MeV protons using the former Los Alamos Meson Physics Facility.[Fn fn4] On the AA6061-T6 alloy, these authors discovered that the hardening precipitates fully dissolve at a dose around 0.2 dpa (displacements-per-atom). They also measured the impact of irradiation on the mechanical properties, discovering that after irradiation at room temperature, both ultimate tensile strength (UTS) and yield strength (YS) reduced by 60 and 80%, respectively, indicating alloy softening after proton irradiation, compatible with the observation that proton irradiation dissolves the hardening precipitates. In experimental support of these claims, the authors presented TEM micrographs of the alloys before and after irradiation and under bright-field TEM (BFTEM): the precipitates were clearly dissolved, and a higher number density of dislocations was noted in their postirradiation BFTEM micrographs (not reproduced here due to copyright restrictions).

We reproduced the results obtained by Lohmann–Sommer et al.^170^ in 1986 on the AA6061-T6, but at higher doses and using the technique of in situ TEM with 1 MeV Kr heavy-ion irradiation at the intermediate voltage electron microscope (IVEM) facility located at the Argonne National Laboratory in Chicago, USA. Heavier ions with energies equal to or below 1 MeV can effectively reproduce the primary knock-on (PKA) energy spectrum that high-energy protons generate in aluminum, as we demonstrated in a previous publication,^34^ and obviously at a much higher dose rate than in space. Figure 10A shows the typical BF-STEM microstructure of the AA6061-T6 alloy, which is composed of Mg–Si-rich solute clusters and Guinier-Preston (GP) zone type II precipitates, known as β″-phase with Mg:Si ≈ 1.^171–173^ In Figure 10B, the microstructure of the alloy after irradiation up to 2 dpa is noted: typical diffraction-contrast attributed to precipitates in Figure 10A is significantly reduced in Figure 10B, and we also noted the nucleation and growth of typical dislocation loops (displacement damage). Some precipitate remnants can still be seen in the micrograph, but the majority of them have dissolved. Obviously, the technology of electron microscopes has evolved quite significantly from the time Lohmann-Sommer performed their research, allowing us to better visualize the damaged microstructure of aluminum alloys with higher spatial resolution.

**Figure 10:**
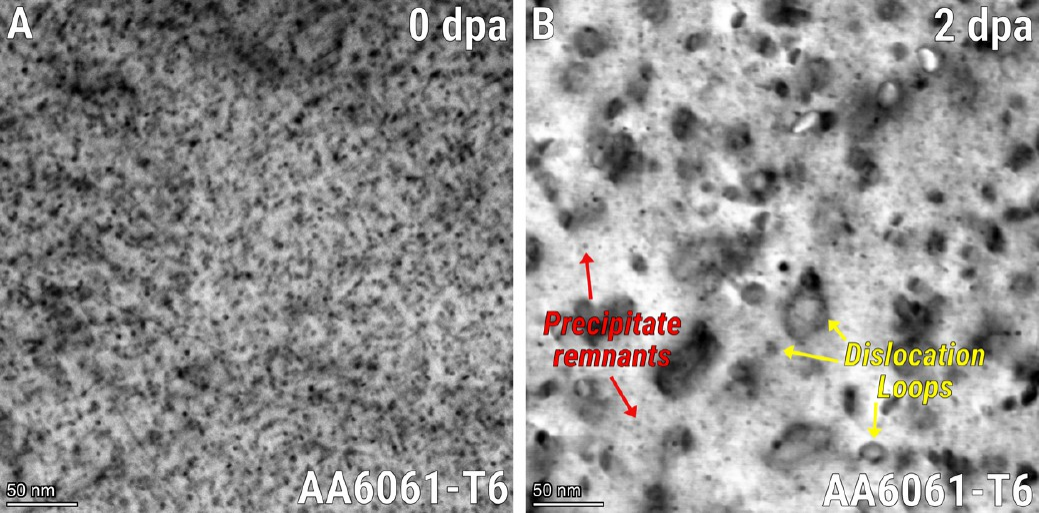
Irradiation effects on AA6061-T6 alloy.BF-STEM micrographs taken closer to the [001]_Al_ zone-axis showing the AA6061-T6 microstructure (A) before and (B) after irradiation up to 2 dpa. Note: Author’s previously unpublished work.

It is important to emphasize that heavy-ion irradiations were also carried out with 2 MeV Au^2+^ and 4 MeV W^3+^ on the commercial AA6061-T6 by Flament et al.^174^ These authors claim that the hardening precipitates in this alloy system suffer only partial dissolution and a slight lattice distortion at doses up to 95 dpa, with complete dissolution only being observed at a dose of 165 dpa. This is a remarkable result, and it is herein reproduced in Figure 11A–D. Nevertheless, it is not clear whether postirradiation screening has been performed at lower doses, such as 0.2 dpa, as for Lohmann–Sommer et al.,^170^ or at 2 dpa, as shown by our ongoing work in Figure 10A,B. The result from Flament et al.^174^ may suggest that, under prolonged irradiation, these phases are able to precipitate back into the microstructure of the AA6061 alloy at higher doses, even if they dissolve at lower doses. It is well-known that energetic particle irradiation drives both interstitial and vacancy diffusion; therefore, it will not be surprising if the β″-phase reprecipitates back during irradiation up to 95 dpa, given the metallurgical system meets favorable thermodynamic conditions. It is noteworthy that the formation of displacement damage-type defects, such as loops and voids, was not documented by Flament et al.,^174^ despite the aggressive irradiation conditions applied and high dose attained. The facilitated formation of dislocation loops (their movement) and voids in aluminum (and alloys) during irradiation is fairly documented throughout the history of nuclear materials.^175–177^ The irradiation conditions chosen by Flament et al.^174^ also depart from the idea of emulating the energetic spectrum of Al PKAs generated by solar wind protons, which naturally was not the major objective of that work.

**Figure 11:**
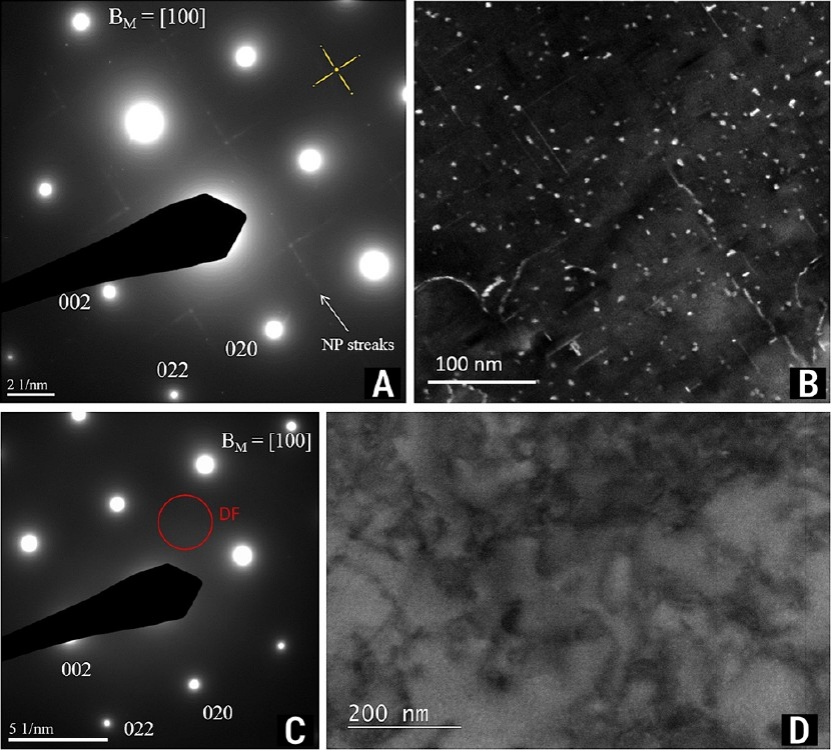
Heavy-ion irradiation of the AA6061-T6 alloy. The diffraction pattern (A) and microstructure (B) of the AA6061-T6 alloy after 95 dpa, showing partial dissolution of hardening precipitates. After 165 dpa, Flament et al.^174^ report on the complete dissolution of precipitates as shown in (C,D). Note: All micrographs in the figure were reproduced with permission from Flament et al.^174^ (Copyright 2017 Elsevier).

In summary, considering studies on the ion irradiation of aluminum alloys, if age-hardenable alloys are to be used in environments where energetic particle irradiation is present (such as the space), one needs to address the survivability of the hardening precipitates to irradiation: if they dissolve after a certain dose, the initial strength of the designed alloy can be lost, thus jeopardizing future long-duration space missions. Displacement damage types such as dislocation loops and the possible formation of voids (and bubbles due to the addition of hydrogen) are also defects to be taken into consideration, possibly leading the alloy to embrittlement (voids) and hardening (dislocation loops).

### Advent of Aluminum Crossover Alloys

In the late 2020s, our research group in Austria carried out an alloy design exploratory research aiming at combining both the high elongation of AA5xxx series alloys (Al–Mg) with the inherent high strength of AA7xxxx series alloys (Al–Zn–Mg). The result of this research was the invention of an entire new class of aluminum alloys, nowadays known as the aluminum crossover alloys.^178–180^ The crossover alloy principle revolutionized alloy design by combining two distinct aluminum alloy classes into a novel alloy with the aim of blending their properties. The 5xxx and 7xxx merge is now under commercialization by the AMAG Austria Metall AG, and it features values of yield strength and elongation approximately in between its two constituent classes.^181,182^

In the process of characterizing the new crossover alloy 5.7, we discovered that one of the major differences between the new alloy and existing commercial aluminum alloys lies in its hardening phase, the T-phase, with chemical formula Mg_32_(Al, Zn)_49_. The role of T-phase precipitates in the crossover alloy 5.7 is to hinder, mitigate, or pin the movement of dislocations in the alloy’s matrix, thus increasing its strength. Figure 12 shows a comparison between the crystal structures (Figure 12A,C) and the *c*-axis two-unit-cell profile (Figure 12B,D) for both the η- and T-phases. A comparison between the T-phase of most commercial aluminum alloys’ hardening precipitates was recently performed by Willenshofer et al.^35^ An intriguing fact about T-phase is that it is a highly concentrated crystal structure with 162 atoms in its unit cell, a scientific fact discovered by Bergmann–Waugh–Pauli^184^ in 1957. This is a significantly higher number of unit cell atoms than the η-phase, for example, which features 12. As shown in Table 3, the β-phase in the AA6xxx series alloy also features 12 atoms in its unit cell. T-phase also presents a larger cell volume, which is 1 order of magnitude higher than the typical aluminum alloys’ hardening precipitates. The presence of the T-phase can be attested using different characterization techniques, such as TEM via diffraction pattern along [001]_Al_, STEM-EDX elemental mapping, and atom probe tomography (APT).^178–180^ Figure 13 shows the characteristic signature of T-phase precipitates in a double-step heat-treated aluminum crossover alloy 5.7 in both peak- and long-aged (or overaged) conditions.

**Figure 12:**
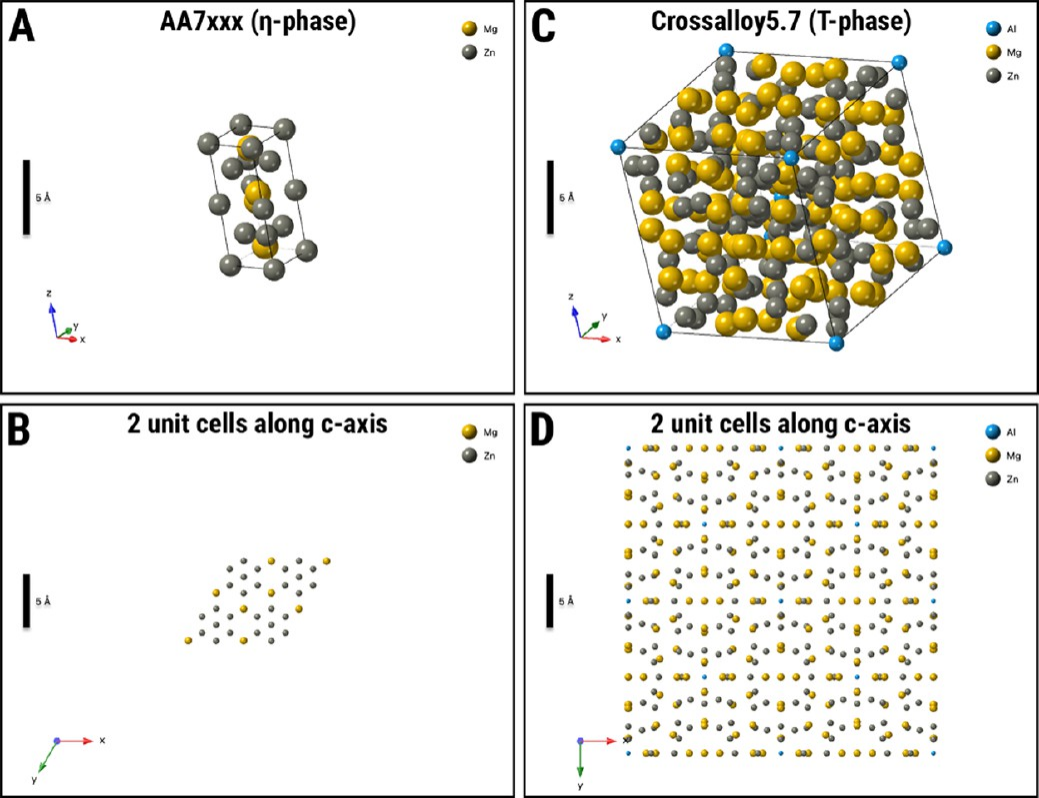
T-phase is the highly concentrated hardening precipitate of the new aluminum crossover alloy 5.7. The crystal structure and 2-unit-cells c-section of the η-phase^183^ in AA7xxx series alloys are shown in (A) and (B). Similarly, the crystal structure and 2-unit-cells c-section of the T-phase^184^ in the aluminum crossover alloy 5.7 are shown in (C) and (D).

**Table 3:** Crystallographic Parameters Comparison between Select Aluminum Alloys’ Hardening (equilibrium) Precipitates

	**chemical formula**	**space group**	**crystal system**	***a*** (Å)	**cell volume** (Å^3^)	**total sites in unit cell**
η-phase^183^	MgZn_2_	*P*6_3_/mmc	hexagonal	5.223	202.371	12
β-phase^185^	Mg_2_Si	Fm3̅m	cubic	6.391	261.040	12
T-phase^184^	Mg_32_(Al, Zn)_49_	Im3̅	cubic	14.160	2839.159	162

**Figure 13:**
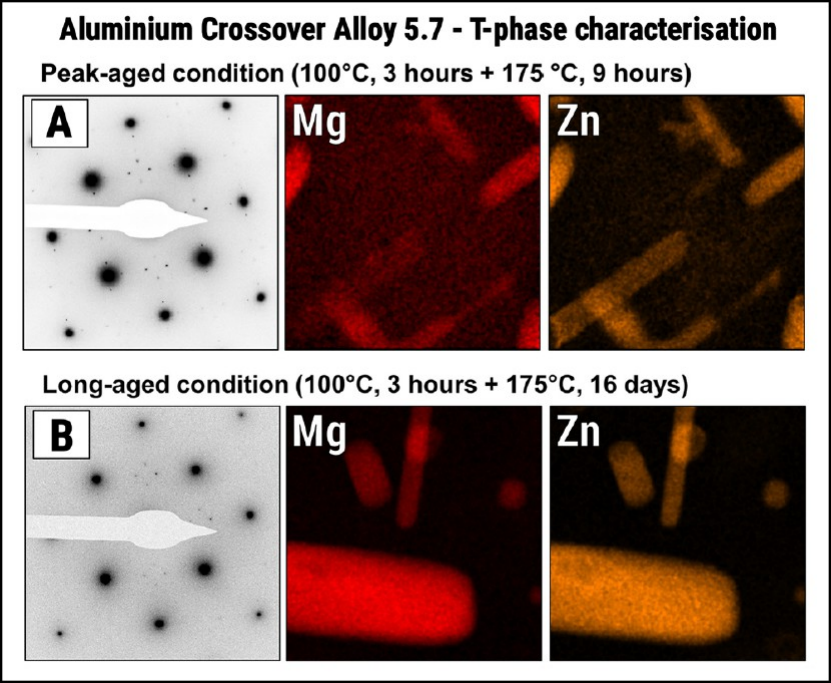
T-phase morphologies in peak- and overaged aluminum crossover alloy 5.7. Characteristic SAED patterns’ signature and STEM-EDX elemental maps showing T-phase precipitates in both double-step (A) peak-aged and (B) overaged conditions. Note: Extracted from Stemper–Tunes et al.^178^ under open access CC-BY-NC-ND license (Copyright 2020 Stemper et al.^178^).

The idea of having highly concentrated and chemically complex precipitates like the T-phase motivated us to pursue ion irradiation studies on the new aluminum crossover alloy 5.7, aiming at investigating their response to energetic particle irradiation environments such as space. This momentum was built upon previous observations at that time reporting that bulk HEAs—which are also highly concentrated and chemically complex metallic alloys—exhibited higher radiation resistance than their elemental constituents and terminal solid solutions.^186–188^ With the idea that T-phase precipitates somehow resemble the typical arrangement of radiation-tolerant bulk HEAs, we wanted to investigate whether this precipitate could survive energetic irradiation bombardment. For that, we prepared a crossover alloy 5.7, but in an overaged state (T7). At this temperature, the T-phase precipitates are in thermodynamic equilibrium with the matrix, and although the alloy is not in its peak-strength condition (T6), we ensured that the majority of phases present were, in fact, equilibrium T-phase (and not, e.g., T′ or T″). Figure 14A–F shows both the microstructure and the diffraction pattern of the aluminum crossover alloy 5.7 under in situ TEM heavy-ion irradiation as a function of the dose. The crystallographic signal attributed to the hardening precipitates is still present on the alloy after a dose of 1 dpa. Although the precipitates are seemingly surviving irradiation, the microstructure of the alloy clearly accumulated a large number density of black-spots, which is a type of displacement damage. The high-radiation resistance of T-phase precipitates against ballistic dissolution is further confirmed in the set of STEM-EDX maps taken before and after irradiation and is shown in Figure 14I,J.

**Figure 14:**
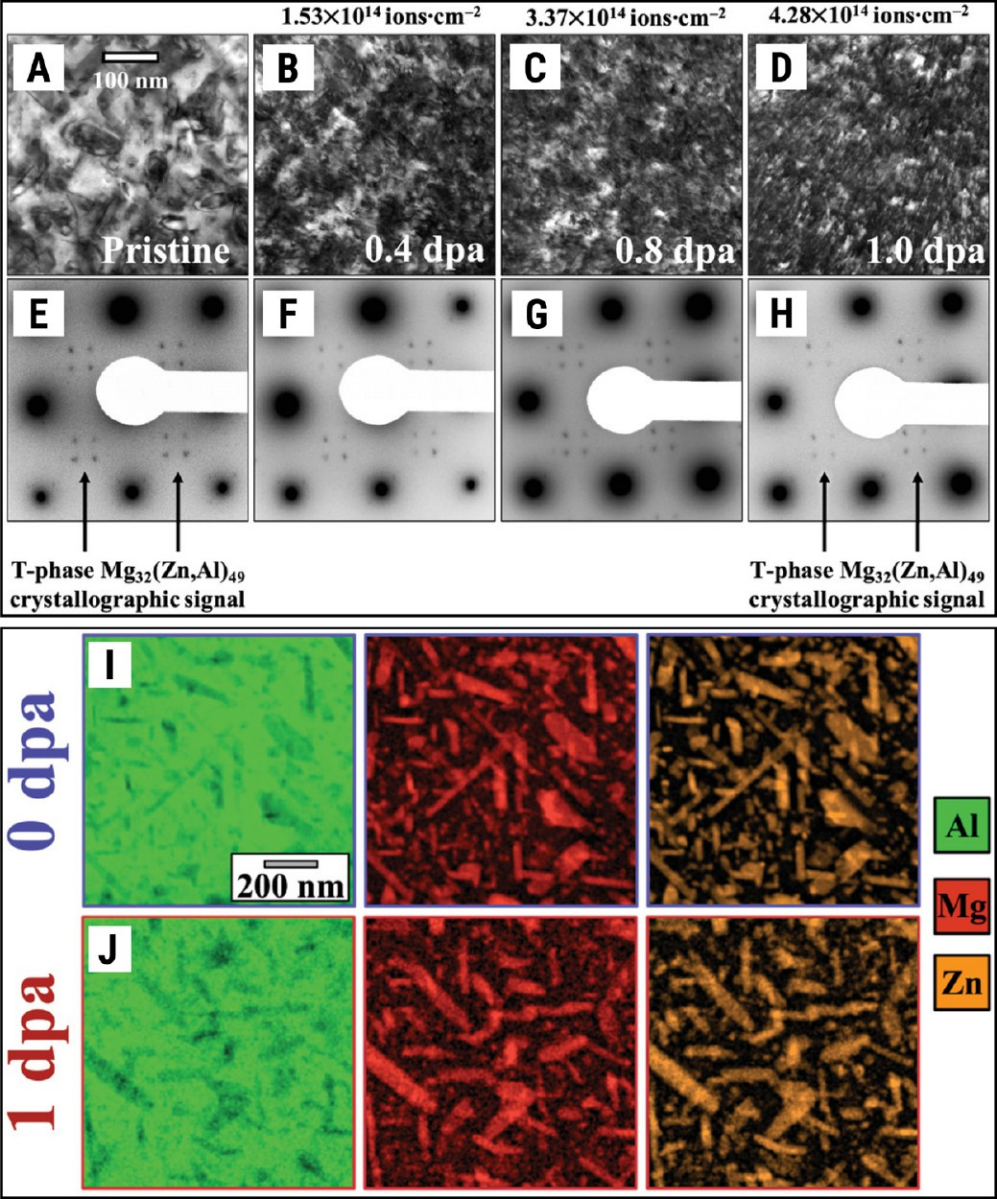
Irradiation response of T-phase precipitates in a novel aluminum crossover alloy 5.7. An in situ TEM heavy-ion irradiation study showing the evolution of the aluminum crossover alloy 5.7 microstructure (A–D) and diffraction pattern along [001]_Al_ (E,F) as a function of the irradiation dose: the accumulation of black-spots is noted in the microstructure, but the precipitates’ diffraction signal is present after 1 dpa. STEM-EDX mapping before and after irradiation also confirms that the T-phase survived 1 dpa. Note: Reproduced from Tunes et al.^34^ under open access in the CC-BY-4.0 license (Copyright 2020 Tunes et al.^34^).

Our work with the overaged aluminum crossover alloy 5.7 has revealed three key design principles for developing radiation-hardened and lightweight aluminum alloys for future long-term space missions where energetic particle irradiation from the Sun is a potential major concern for materials:

1. Hardening precipitates must remain stable under irradiation. Ideally, their volume fraction should be high to assist with defect recombination. The T-phase was confirmed to present a higher volumetric fraction than the commercial AA6061 alloy.^34^
2. The T-phase showed notable resilience when compared with previous results published on the AA6061-T6 alloy featuring β″ precipitates dissolving at a dose around 0.2 dpa.^170^ Thus, it is a suitable hardening precipitate to compose aluminum alloys for application in extreme environments.
3. Radiation-induced displacement-type defect accumulation—such as loop formation—should be minimized. Future aluminum alloys with refined grain structures could offer more defect sinks via grain boundaries, thus reducing internal damage build-up and accumulation.

While the origins of the T-phase’s exceptional resistance were not fully understood in our previous study,^34^ its presence in high volumetric fraction and chemically complex structure appears beneficial in radiation environments. Future work should investigate how heat treatments, precipitate morphology, and irradiation-driven changes to minor intermetallics (e.g., Cr-, Fe-, and Mn-rich phases) influence the alloy’s stability. Techniques like atom probe tomography and mechanical postirradiation testing will be critical in advancing this research area.

### Are Ultrafine-Grained Aluminum Alloys the Future Space Materials?

Synthesis of new UFG metallic alloys via methods of severe plastic deformation (SPD) became a key topic in metallurgical research in the last two decades. The foundational and methodological works on UFG processing were reviewed by several seminal papers where UFG aluminum alloys occupy the center of attention.^189–192^ Up-to-date, UFG aluminum alloys have demonstrated potential for applications in the fabrication of MEMS components,^193^ high-temperature structural parts,^194^ aerospace and defense systems,^195^ and advanced welding technologies.^196^ Recent developments, challenges, advanced phenomena, and state-of-the-art in UFG aluminum alloys (and other UFG materials) have been discussed in detail by Langdon et al.,^197^ Huang et al.,^198^ Chinh et al.,^199^ and Harsha et al.^195^ The application of UFG metallic alloys in extreme environments presents several distinct advantages that are being exploited for future real applications. The idea of grain-refining metallic alloys toward the nanoscale generates microstructures with an extraordinarily large number of sinks for point defects; thus, these sink sites contribute to reducing the build-up and accumulation of irradiation-induced defects, promoting fast recombination and recovery.^200^ For example, it has been demonstrated that nanocrystalline tungsten exhibits higher radiation tolerance than its coarse-grained (CG) counterpart.^201^ Building on this, UFG refractory HEAs are now revolutionizing the development of materials for thermonuclear fusion reactors.^202^

Our research group initiated research on UFG aluminum alloys, aiming to investigate their potential space applications, and we explored both their thermal and irradiation performances. Building upon the previous knowledge of the crossover alloy 5.7 under irradiation,^34^ Patrick Willenshofer's PhD work has led to the first-time synthesis of a UFG aluminum crossover alloy in the quinary system comprising Al–Mg–Zu–Cu–Ag.^35,36,203,204^ Figure 15A,B show the BFTEM micrographs of the CG and UFG aluminum crossover alloy microstructures, respectively. To produce a UFG microstructure, Willenshofer applied the SPD technique of high-pressure torsion (HPT). Willenshofer et al.^203^ discovered that a heat-treatment of 233 °C promotes T-phase precipitation on the UFG microstructure of the alloy at both transgranular and intragranular positions. T-phase precipitates at these specific site-dependencies inhibit the early recrystallization characteristic of UFG aluminum alloys (see the review of Valiev and Landgon^190^). In this way, this UFG aluminum crossover alloy is a potential material for application in satellites in LEO, as its UFG microstructure is thermally stable up to 200 °C and more. The master's thesis of Sandra Gonzaga^37,205^ in our group has shown, for example, that a UFG version of the commercial AA6061 is not as thermally stable as the UFG aluminum crossover alloy.

**Figure 15:**
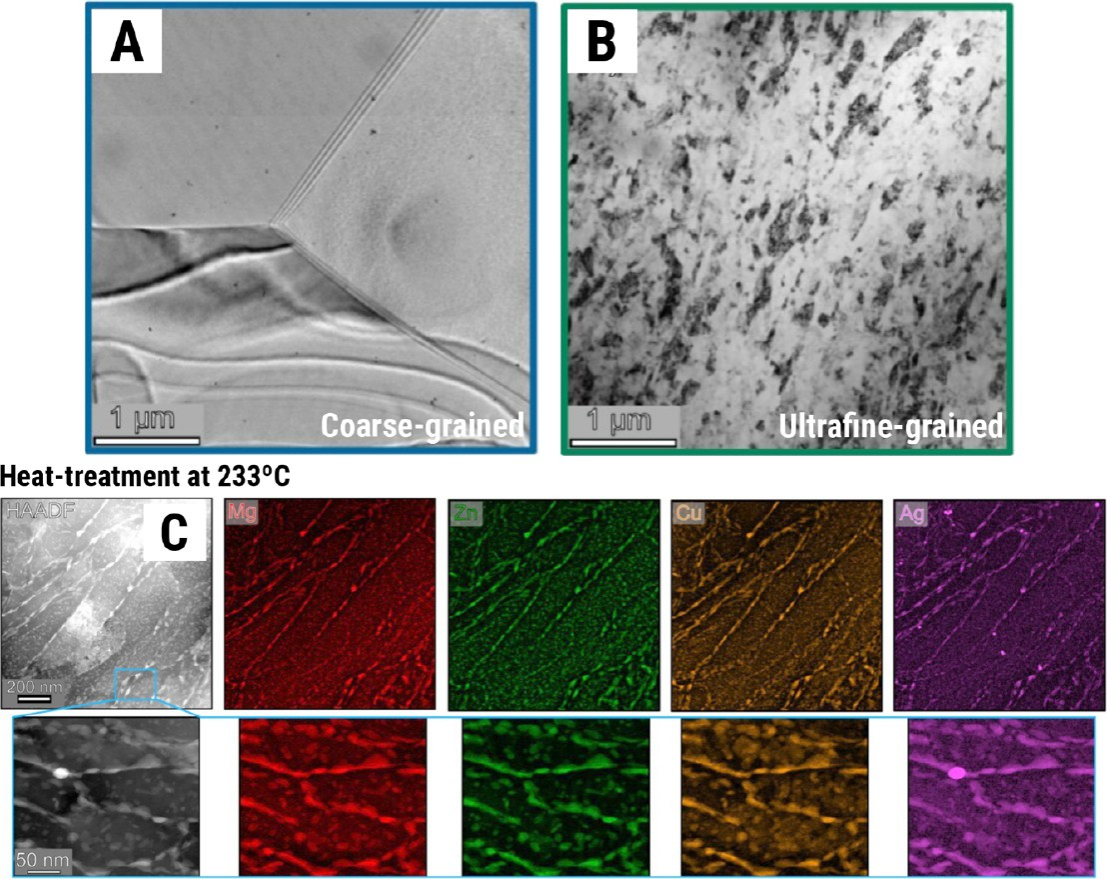
Novel UFG aluminum crossover alloy 5.7. The (A) CG and (B) UFG aluminum crossover alloy (AlMgZnCuAg). (C) Set of STEM micrographs and EDX elemental maps showing T-phase precipitation at transgranular and intragranular sites. Note: Reproduced from Willenshofer et al.^203^ under open access in the CC-BY-4.0 license (Copyright 2023 Willenshofer et al.^203^).

Willenshofer et al.^203^ investigated the thermal stability of the novel UFG aluminum crossover alloy using the technique of in situ TEM heating, and the results are shown in Figure 16. T-phase precipitation starts at around 200 °C, and the UFG microstructure can be considered stable up to 300 °C. We discovered that T-phase precipitation along the grain boundaries promotes their pinning (see Figure 15C), delaying recrystallization to higher temperatures. This is a phenomenon observed particularly in the new UFG aluminum crossover alloy system: commercial aluminum alloys with UFG microstructure are prone to recrystallize at approximately 200 °C.^37,190,205^ The fact that this new UFG aluminum crossover alloy shows markedly thermal stability, it could be considered for application in space, especially in LEO, where thermal fluctuations due to shadow and sunlight exposure are major concerns, as it can increase the temperature of aluminum up to 180–200 °C, as explained in the previous section.

**Figure 16:**
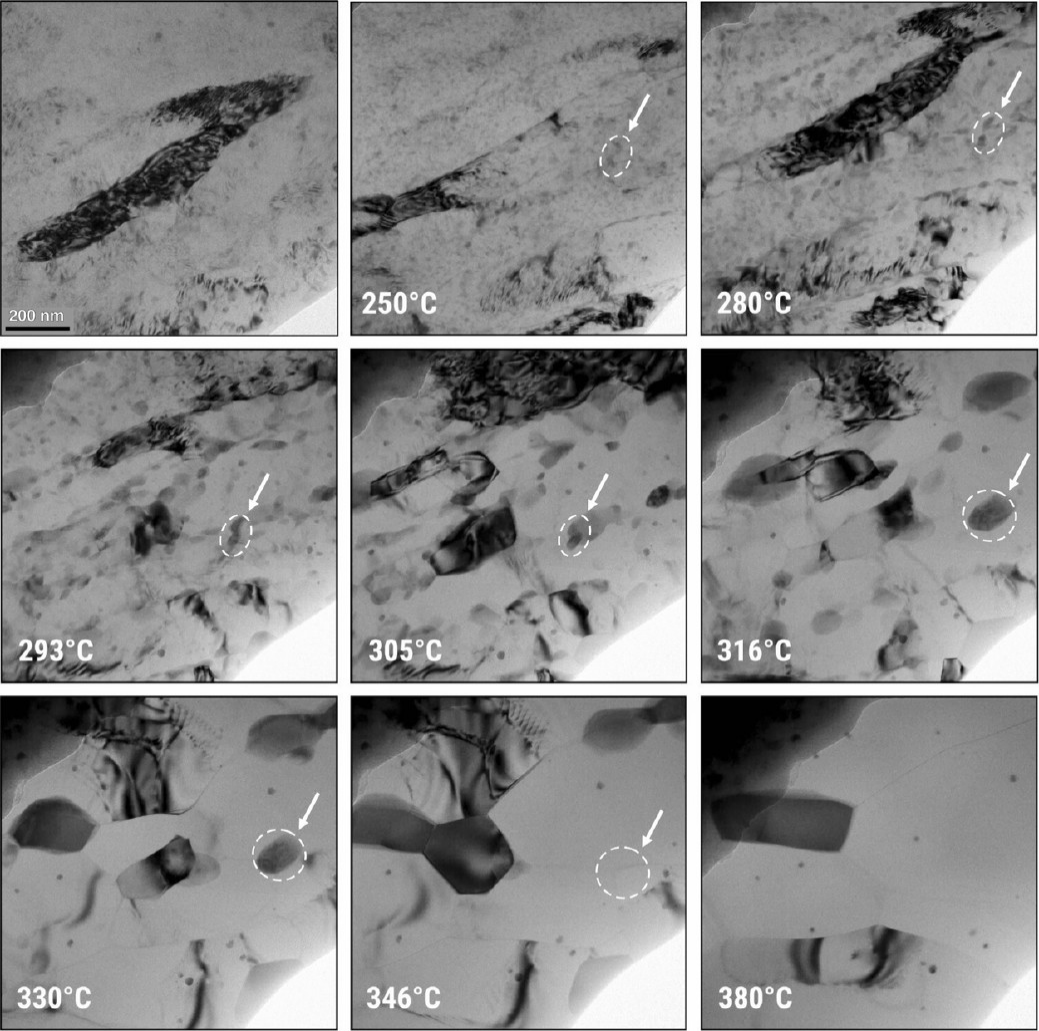
A stable UFG aluminum alloy up to 300°C. Application of the in situ TEM heating technique to investigate thermal-induced recrystallization of the novel UFG aluminum crossover alloy AlMgZnCuAg. Precipitation of the T-phase starts at around 200 °C, and the grain microstructure is seemingly stable up to nearly 300 °C. White dashed circle marks nucleation and growth of select T-phase precipitates. Note: Reproduced from Willenshofer et al.^203^ under open access in the CC-BY-4.0 license (Copyright 2023 Willenshofer et al.^203^).

A question that remains on the new UFG aluminum crossover alloy is: How does it behave under irradiation? As previously mentioned, most conventional age-hardenable aluminum alloys lack sufficient resistance to SEP radiation. Their hardening phases tend to dissolve, and displacement damage accumulated in the matrix severely impairs the performance. In a recent preprint, we tested the irradiation resistance of the UFG aluminum crossover alloy and found that up to 24 dpa, T-phase precipitates are stable, and the UFG microstructure neither forms nor accumulates dislocation loops within its grains.^35^ In this latter work, we attempted to correlate the T-phase with a giant unit cell and highly negative enthalpy of formation with the observed high-radiation resistance.

As demonstrated above, T-phase precipitates resist ballistic dissolution under energetic particle bombardment. Combined with nanometer-scale grain sizes, this strategy imparts high radiation resistance to UFG aluminum crossover alloys. An alternative, counterintuitive design concept was proposed by Wu et al.,^206^ who investigated the role of excess vacancies in the low-temperature decomposition of the supersaturated solid solution in a UFG AlCuSc alloy—a modified version of the conventional AA2xxx series. They showed that when the population of SPD-induced excess vacancies is further increased by superficial ion bombardment and cryogenic processing, they act to stabilize Cu–Sc-vacancy complexes. This mechanism prevents and/or delays undesirable precipitation during subsequent high-temperature annealing while retaining high strength. While the UFG aluminum crossover alloy concept introduced by our group relies on T-phase precipitates to provide high strength, high irradiation, and thermal stabilities, deliberately exploiting irradiation-induced excess vacancies to suppress precipitation could represent another promising pathway for aluminum alloy design in space applications, but one must consider that precipitation-free UFG aluminum alloys may suffer recrystallization at temperatures ≤ 200 °C. Although these concepts offer promising pathways for designing next-generation radiation-resistant materials for space applications, bulk-scale manufacturing of UFG aluminum alloys still remains a major metallurgical challenge.

## Future of Aluminum Alloys as Space Materials

The following provides a concise overview of current challenges in space materials, with particular focus on unresolved issues, potential approaches, and future research directions for aluminum alloys in space applications. While not exhaustive, this outline reflects an open exercise in scientific practice and curiosity.

### Aluminum Activation under the Bombardment of Protons

When exposed to medium- or high-energy protons, aluminum undergoes nuclear reactions leading to activation. Of particular relevance for space applications are reactions in which aluminum captures a proton and emits neutrons or γ rays. As shown in Figure 17, the stable isotope of aluminum exhibits significant cross sections for several nuclear reactions in the energy range of relevance for solar wind protons (most probably occurring with *E* ≈ 1–10 MeV, but extending up to GeV^96^).

**Figure 17:**
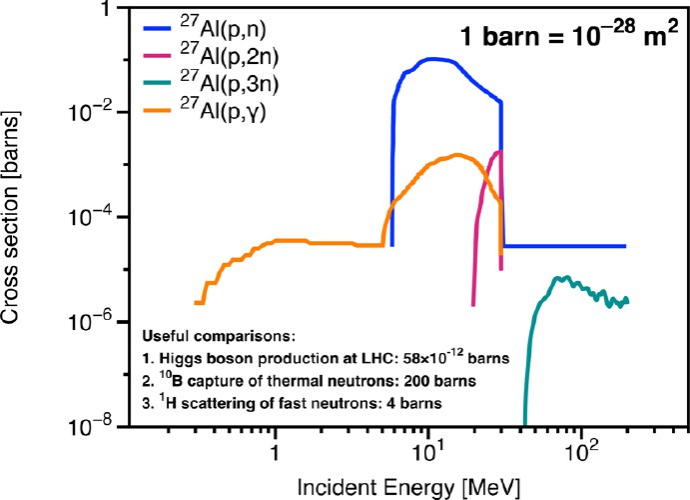
Aluminum activates under proton irradiation. Cross section for the following nuclear reactions ^27^Al­(p,n), ^27^Al­(p,2n), ^27^Al­(p,3n), and ^27^Al­(p,γ). Note: Public data replotted from the public domain Evaluated Nuclear Data File (ENDF) database.^207,208^

Here, it is important to clarify the definition of cross-section and the order of magnitude of such nuclear reactions caused by proton bombardment of aluminum. In high-energy and nuclear physics, cross-section is expressed by the unit of “barn,” which quantifies the likelihood (i.e., probability) of interaction and/or reaction between particles. One barn corresponds to an area about that of a uranium nucleus. The cross-section values presented in Fig. 17, at a glance, may appear as “low values” when compared with other nuclear reactions. For example, the nuclear fission reaction of ^235^U nuclei under thermal neutron bombardment has a cross-section of around 585 barns (so a very high probability of occurrence). Conversely, when ^12^C nuclei are bombarded with neutrons, γ rays are produced, and such a reaction has a cross-section of around 0.035 barns.

Although neutron and gamma emission from aluminum under proton bombardment are considered events of low probability, they may cause problems to human crew health on long-term/long-duration space missions such as those planned for space exploration and extraterrestrial settlement. Neutrons and γ rays pose significant risks to space explorers’ health, adding to the hazards already presented by solar wind protons and CGRs.

Recently, the field of metal–matrix composites (MMCs) has given an insight into future aluminum-based space composites that may address the problem of neutron generation due to solar wind proton bombardment. The idea is to dissolve boron carbide (B_4_C) particles within aluminum alloys’ matrices.^209–214^ This ceramic material is known to be highly neutron absorbent, which can capture energetic neutrons from (p,n) nuclear reactions. In Figure 18, I reproduce (with permission from Elsevier) an extraordinary MMC synthesized and characterized by Chao et al.^209^ using the AA2024 alloy with 25 vol % of B_4_C. The idea I have is shown in Figure 18A,B. If a neutron is born in the aluminum alloy matrix, it will likely be captured by the nearest B_4_C. A highly neutron-reflective material (such as Be, C, W, etc.) could coat the inner side of the MMC to guarantee that no born neutron escapes the material. This coating will reflect the neutrons back to the MMC microstructure, increasing the probability of their absorption. The level of B_4_C to effectively attenuate the neutrons could be calculated using known neutron transport codes in future projects (such as MCNP, openMC, SERPENT, etc). Chao et al.^209^ comment on an improvement of these MMCs under projectile impact; therefore, future space MMCs could address both radiation shielding and micrometeoroid impact.

**Figure 18:**
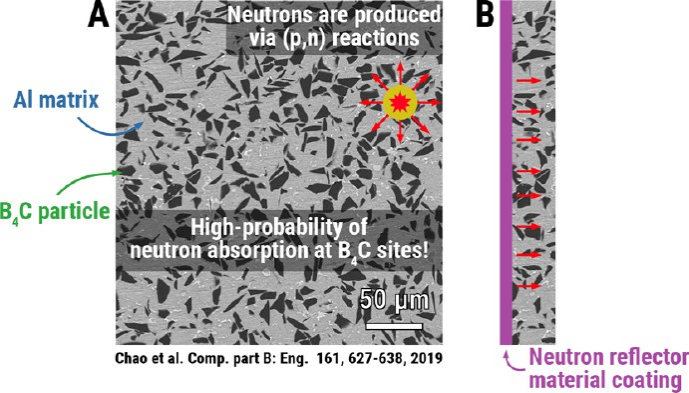
Protecting crew against neutrons with advanced MMCs. Aluminum alloy composites with ceramic particles like the B_4_C have the potential to protect human crew from neutron irradiation after solar wind proton bombardment. (A) The idea is that the neutrons are born in the microstructure of the aluminum matrix and are absorbed by the nearest B_4_C particle. (B) A coating made of a highly neutron-reflective material can be manufactured with the MMC, so that, if neutrons escape the MMC microstructure, it can be backscattered, thus increasing the probability of capture. Note: The micrographs in (A) and (B) were extracted and modified with permission from Chao et al.,^209^ it shows the AA2024 matrix with B_4_C particles at 25 vol % (Copyright 2019 Elsevier).

The case of gamma-ray generation is a little more complex, as high-density materials are necessary to properly shield this form of ionizing radiation.^89^ The coating idea in Figure 18B could serve the dual purpose of reflecting neutrons and also absorbing gamma-rays. Tungsten is a highly effective neutron reflector and high-attenuating gamma material. The choice of this coating must address, nevertheless, the strength-to-weight requirements of space materials. Recent work performed by our group on the deposition of diamond-like-carbon coatings onto AA6061-T6 is a possible route for research in this context.^215^ Nevertheless, research on aluminum-based MMCs for space applications is expected to expand in the coming years, driven by the need to address key challenges in space materials.

### Equivalence between Radiation Protection Dose Units and Material Dose

The community of radiological protection has two distinct definitions to express radiation dose, and they must be carefully noted:

- Absorbed dose (*D*): Express the total absorbed energy of radiation in J kg^–1^. One Gray (1 Gy) is equal to 1 Joule of absorbed radiation per unit of mass.
- Dose equivalent (*H*): Quantifies the potential for stochastic health effects from ionizing radiation, such as the likelihood of developing cancer or hereditary disorders. The unit is Sievert, and it is defined by the product of the radiation weighting factor (*w*_R_) with the absorbed energy in Joules per kg. One Sv = *w*_R_ J kg^–1^.

In the field of space radiation protection, material radiation dose is typically expressed as absorbed dose in Gy. This is often observed for shielding calculations for both structural materials and electronic components.^216,217^ While this approach is valid for both radiation protection and materials science, the radiation effects community—focused on damage and microstructural defect analyses—uses two different measures: particle fluence (particles cm^–2^) and displacements per atom (dpa). Establishing a correlation between absorbed dose (Gy), equivalent dose (Sv), and radiation damage effects in materials’ microstructures (dpa) would enable faster, more direct comparisons of radiation exposure levels between human health contexts and material defect generation.

Monte Carlo codes such as SRIM^218^ are commonly used to calculate radiation damage in dpa,^219,220^ but can also estimate the energy transferred to aluminum recoils as a function of penetration depth. This is the energy absorbed by a target as a result of an incident energetic ion. As shown in Figure 19, 2.5 MeV protons penetrating aluminum progressively transfer their kinetic energy to aluminum recoils. Absorbed dose can be (“tallied”) calculated under similar proton irradiation conditions by computer codes like the Monte Carlo N-particle (MCNP)^221^ and the GEANT-4.^222^ Such calculations could support future correlations between the absorbed dose (Gy) and radiation damage dose (dpa). Unifying or directly correlating absorbed dose (Gy) with radiation damage dose (dpa) is essential for future space missions, as it will directly inform and advance the design of next-generation space materials in the face of the existing literature. Experts in nuclear transport codes could contribute to the solution of this problem.

**Figure 19:**
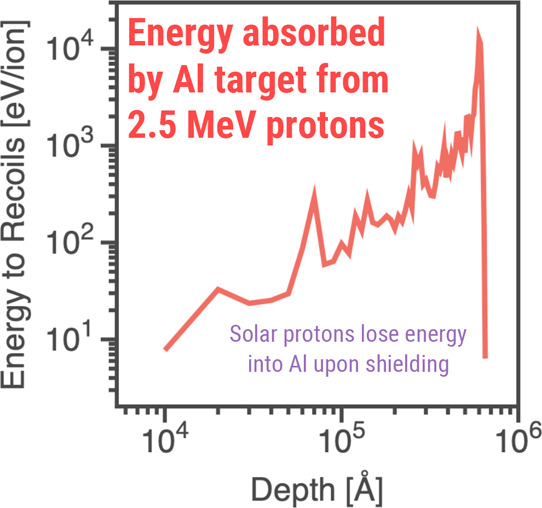
Absorbed dose by aluminum bombarded with one 2.5 MeV proton. SRIM’s estimation of energy transferred to recoils due to the bombardment of 2.5 MeV protons.

### Hydrogen Embrittlement May Be a Problem for Space Materials

As discussed earlier, energetic particle radiation can dissolve hardening phases in aluminum alloys, causing softening, while also generating excess voids and dislocation loops, leading to irradiation hardening. In spacecraft and satellites, aluminum serves both as a structural and shielding material against harmful radiation, particularly solar protons that must be stopped within the spacecraft skin. Over time, this shielding role inevitably results in hydrogen accumulation within the aluminum lattice. The effects of the presence of monatomic hydrogen in the microstructure of metals, alloys and materials is a phenomenon known as hydrogen embrittlement, in which the discovery, completes 150 years in 2025.^223–227^ In certain aerospace-grade 7xxx series aluminum alloys, around of 1–2 weight parts per million (wppmH) of hydrogen have been reported to markedly reduce elongation-to-fracture (plasticity).^228,229^ Both the metallurgical and chemical aspects of hydrogen embrittlement have been independently discussed in three recent review papers, which are herein referred to the informed reader.^226,227,230^

Figure 20 shows hypothetically how different degradation forces can change the initially designed mechanical properties of an aluminum alloy. For long-duration space missions, hydrogen embrittlement may pose a problem for future space materials, especially aluminum alloys. Recent strategies to mitigate this effect focus on immobilizing hydrogen within the microstructure of aluminum alloys through phase engineering. Zr-^228^ and Sc-bearing^229^ precipitates can effectively trap hydrogen, potentially delaying and/or reducing the deleterious effects of hydrogen embrittlement. However, their stability under energetic particle irradiation remains unknown, raising the question of whether precipitation-based approaches are truly reliable for resisting hydrogen embrittlement in space environments. UFG aluminum alloys could effectively mitigate the deleterious effect of hydrogen, as the presence of a large number of sinks (grain boundaries) could contribute to removing hydrogen atoms from the microstructure. Nevertheless, their fabrication on a bulk scale and in large quantities remains a challenge.

**Figure 20:**
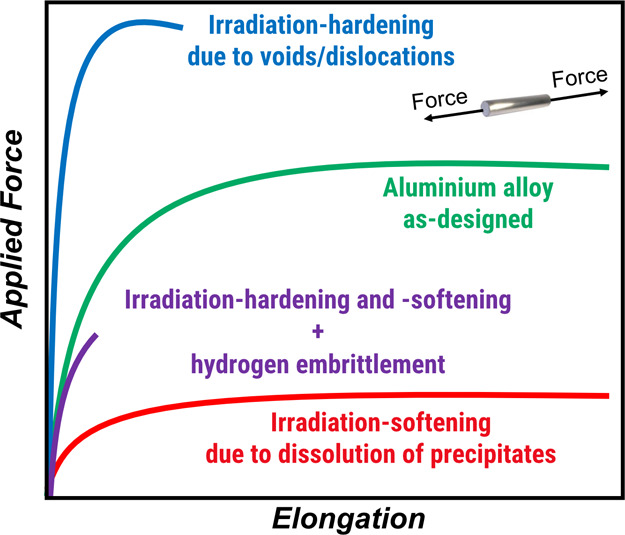
Hydrogen embrittlement in space? Aluminum serves as shielding to solar wind protons, which stop within the material. Accumulation of hydrogen in aluminum eventually contributes to hydrogen embrittlement. Whether radiation damage in conjunction with hydrogen embrittlement impacts the microstructure and mechanical properties of aerospace-grade aluminum alloys is not yet known.

### Fabrication of Bulk UFG Aluminum Alloys in Industrial Scales

The large-scale and mass-production fabrication of UFG alloys remains a challenge for metallurgy.^231^ SPD fabrication techniques are still limited to laboratory-scaled samples, which limits the application of UFG aluminum alloys as future space materials, although they are potential candidates that may offer superior radiation and hydrogen embrittlement resistances. In combination with modern welding/joining technologies, perhaps, small UFG aluminum alloy samples could be joined to form a large-scale sheet or billet that could be shaped into a structural material for a spacecraft.

### High Strain Rates and Micrometeoroid Impact

As previously mentioned, the impact of micrometeoroids on the microstructure of aluminum alloys can occur at velocities ranging from 4 to 51 km s^–1^, with an estimated average velocity of around 20 km s^–1^ (see refs 33, 68, and 69 ). The influence of high strain rates on the microstructure of aluminum and its alloys upon impact remains insufficiently understood and warrants further investigation to clarify the deformation mechanisms in this regime.

The seminal work of Gray III at the Los Alamos National Laboratory may shed light on what happens to aluminum alloys under the extreme dynamics posed by micrometeroid impacts. Gray investigated the shock-loading impact of tungsten and molybdenum projectiles into the Al-4.8 Mg (wt.%) alloy at velocities around 1 km s^–1^.^232^ The resulting microstructures are shown in Figure 21A–C. He discovered that under the impact of high-speed projectiles, aluminum alloys undergo deformation via twinning, which is particularly attested by the presence of twin-plates under both BFTEM and DFTEM as well as satellite spots in the diffraction pattern. This observation is counterintuitive as twinning is an uncommon deformation mechanism in face-centered cubic (fcc) metals and alloys, particularly in aluminum, which has a high stacking-fault energy, preventing the formation of twin embryos. Work remains to be done on the effects of higher velocities impacts in the microstructure of aerospace-grade aluminum alloys and other variants.

**Figure 21:**
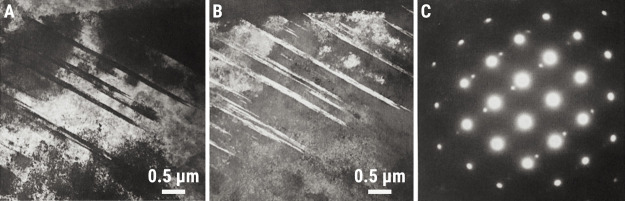
High-strain shock-loaded Al-4.8Mg wt.% microstructure. (A) BFTEM, (B) DFTEM, (C) SAED pattern of the shock-loaded microstructure of the Al-4.8Mg alloy showing deformation-induced twinning. Note: Extracted, repositioned, and reproduced with permission of Gray III^232^ (Copyright 1988 Elsevier).

In addition to shock-loading and recovery experiments, cold-spray and additive manufacturing are interesting avenues to possibly investigate the impact of high-speed projectiles on aluminum substrates.^233^ Some experimental observations of cold-spray particles’ impact onto aluminum substrates reassemble the craters posed by micrometeroids and space debris, as shown in Figure 4. Investigating and understanding the mechanical behavior of aluminum alloy under high-strain rates with the methods of electron microscopy, especially S/TEM, is of paramount importance to design future aluminum alloys for space missions.

### Emerging Topics: Cluster-Hardenable Aluminum Alloys

Cluster-hardenable aluminum alloys derive their strength from the formation of fine solute clusters and precipitates during thermal treatment. Philip Aster in our group is currently leading efforts to investigate cluster-hardenability of novel aluminum crossover alloys in the metallurgical system comprising Al–Mg–Zn–(Cu).^234^ He has recently demonstrated that the addition of Cu was found to markedly influence clustering and precipitation behavior, increasing number density, promoting T-phase precursors, and altering the cluster chemistry from within. APT experiments revealed that Cu promotes the formation of smaller clusters/precipitates at early aging stages, stabilizing the microstructure and suppressing coarsening. Long-term aging (LTA) at 60 °C for 42 days produced an outstanding synergy of yield strength (≈400 MPa) and elongation (≈17%), surpassing conventional short-term treatments in ductility while retaining competitive strength. This improvement was linked to enhanced resistance to dynamic recovery, attributed to the fine and closely spaced cluster/precipitate distribution. These results are shown in Figure 22.

**Figure 22:**
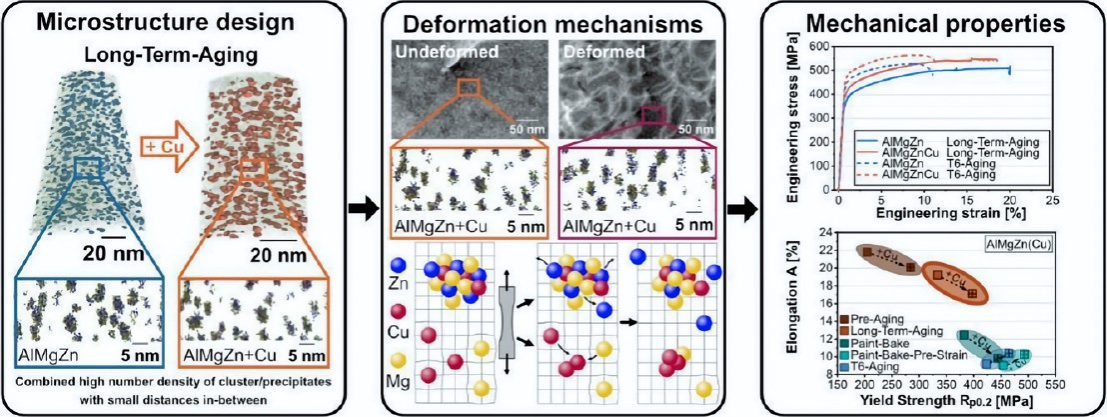
Cluster-hardened aluminum crossover alloy. Aluminum alloys can be hardened with clusters, which are precursor phases of larger precipitates. These clusters can form in the aluminum crossover alloy Al–Mg–Zn–(Cu) system with long-term heat-treatment at temperatures as low as 60 °C, therefore reducing the level of complexity for manufacturing such alloys in loco in space. After cluster-hardening, some select alloys exhibit yield strength around 500–600 MPa with concomitant high elongation, better than some well-known aerospace grade systems (AA7xxx). Note: Reproduced with permission from Aster et al.^234^ under open access in the CC-BY-4.0 license (Copyright 2025 Aster et al.^234^).

The work of Aster et al.^234^ has deep implications for the future of space materials. Producing strong yet ductile alloys with low-temperature heat-treatment addresses a very important requirement that space materials must fulfill: in-space manufacturability. In extraterrestrial settlements, such as in a future lunar base, the directed energy from the Sun is a way to establish a metallurgical facility in space for assisting heat-treatment, and possibly, even melting of aluminum alloys. Nevertheless, so far, combined radiation damage, shielding, and hydrogen embrittlement studies in cluster-hardenable aluminum alloys are scarce, if not inexistent.

### Emerging Topics: Self-Healing Aluminum Alloys

Self-healing materials represent a recent research frontier in materials science that has gained attention across multiple scientific disciplines. When a material experiences damage—either mechanical, thermal, or radiation-induced—and its microstructure is capable of autonomously “curing” the consequences of the damage, the material is said to exhibit a self-healing capability. In this context, the concept of self-healing is a highly desirable property for space materials. Metals and alloys, polymers, and ceramics are currently being designed to feature self-healing against multiple types of external degradation forces.^235–239^

A new class of polymers with self-healing properties against space radiation was recently developed.^240,241^ Polymers occupy a great portion of the space materials industry, yet the fuselage and structural parts of spacecrafts, satellites, and probes still rely on metallurgy, primarily the aluminum alloys. It is also important to emphasize that the mechanisms governing radiation damage in metals and alloys are considerably more complex than polymers,^242^ thus strategies to make aluminum alloys feature self-healing capabilities to energetic particle irradiation are still pending further investigation, although related research directions have been initiated.^243,244^

Given that the space environment imposes multiple degradation forces operating in synergy, future self-healing aluminum alloys will need to demonstrate autonomous repair capability not only against energetic particle radiation but also against corrosion, micrometeoroid impacts, thermal cycling, and other forms of damage. It is an incommensurable challenge that now opens opportunities for high-risk/high-gain scientific projects. Roger Lumley recently reviewed and introduced the major concepts underlying self-healing in aluminum alloys^243^ relies on *“controlled microstructural changes* [mainly via precipitation] *so that they can respond to service conditions.”* The work of Lumey remains nonexhaustive, and the study of self-healing phenomena in aluminum alloys is still in its early stages of development. An integrative approach addressing multiple degradation mechanisms acting in synergy is still pending. UFG aluminum alloys offer a natural strategy to self-heal excess defects generated by events of irradiation, but not necessarily address all these multiple degradation mechanisms acting together. I anticipate that self-healing aluminum alloys (with a nice acronym, SHAA) will constitute the future of materials for space exploration and extraterrestrial settlement.

### Emerging Topics: Artificial Intelligence for Space Materials Research

The field of space materials present degradation forces that could largely benefit from computer simulations and modelling amid predictions of materials’ behaviour in extreme conditions. Furthermore, the synthesis of novel alloys and materials under space conditions differs fundamentally from terrestrial manufacturing, as microgravity and other unique space-specific factors significantly alter solidification behavior and microstructural evolution.^245,246^ Although computational materials science is not an emerging topic in this context, as numerous works have already addressed on the activity of metallurgy in space conditions, the recent advent of artificial intelligence (AI) exhibits an extraordinary potential to revolutionize space materials research.

AI is emerging as a transformative tool in materials research, shifting the paradigm from (almost pure) empiricism toward predictive and autonomous discovery. The role of AI in the future of materials research was recently discussed in a review paper by Milad Abolhasani and Keith A. Brown.^247^ These authors concluded that AI poses a paradigm shift in the very foundations of the scientific method. By exploiting large data sets from both experimental characterization and computational modeling, AI could facilitate the identification of hidden structure–property-processing relationships that are otherwise inaccessible through conventional approaches. Machine learning (ML) algorithms already enable accelerated screening of vast compositional and processing spaces, supporting the inverse design of alloys, ceramics, and composites with targeted functionalities. In combined experimental and computational works, the group of Professor Dermircan Canadinc recently demonstrated that ML has an extraordinary potential to predict and assist the discovery of new alloys with high-ductility/high-strength, high-thermal conductivity, and even predict totally new shape memory alloys.^248–250^ Furthermore, autonomous research platforms that integrate AI with robotics and high-throughput experimentation offer the prospect of closed-loop optimization, substantially reducing development times for novel materials.

Beyond discovery, AI plays a pivotal role in integrating knowledge across multiple scales and domains, and these facts could largely benefit the entire research and development of space materials. Advanced data analysis methods can extract subtle features from microscopy and spectroscopy data sets, enabling real-time feedback in in situ experiments and providing unprecedented insights into microstructural evolution of materials subjected to the extreme conditions found in space. In industrial contexts, AI may contribute to process optimization, quality assurance, and sustainability by predicting the impact of recycling routes, impurity levels, or processing conditions on final material performance. In this context, AI has the potential to evolve into autonomous materials science laboratories, enabling fully automated manufacturing and recycling under extraterrestrial conditions in future human colonies.

Regarding the application of AI methods to aluminum alloys, I believe that the combination of increasing supercomputing power and the vast data sets accumulated over nearly two centuries of research will enable AI to support metallurgists in the complex task of recycling space junk and debris. As most defunct satellites, spent rocket components, and aging spacecraft are composed of high-strength aerospace alloys such as AA2xxx and AA7xxx, AI could integrate and process data from across the materials science domain—including thermodynamic modeling (e.g., CALPHAD, Pandat, molecular dynamics, density functional theory), casting and solidification, thermal processing, and microstructural analysis (SEM, S/TEM, APT, etc.)—to design a recycled space alloy (potentially a new aluminum crossover 2.7!). Such an alloy would need to fulfill stringent space requirements, including resistance to radiation damage, high strength and impact resilience, corrosion resistance, and protection against hydrogen embrittlement: therefore, although AI is an emergent power, human-based experimentation (and curiosity!) will still rule in this context for quite some time. This concept is a simple brainstorming, and it is illustrated in Figure 23. In this context, achieving sustainability and circularity in metallurgy—as recently envisioned by Professor Dierk Raabe^251^—strongly relies on examining the microstructure of metallic alloys and their recycled variants at multiple length scales (macro, micro, nano, and pico-meter). The combination of AI and materials science is indispensable for decoding and validating numerous recycled alloy concepts in a timely manner, perhaps ultimately culminating in the intelligent design of novel space-grade aluminum alloys.

**Figure 23:**
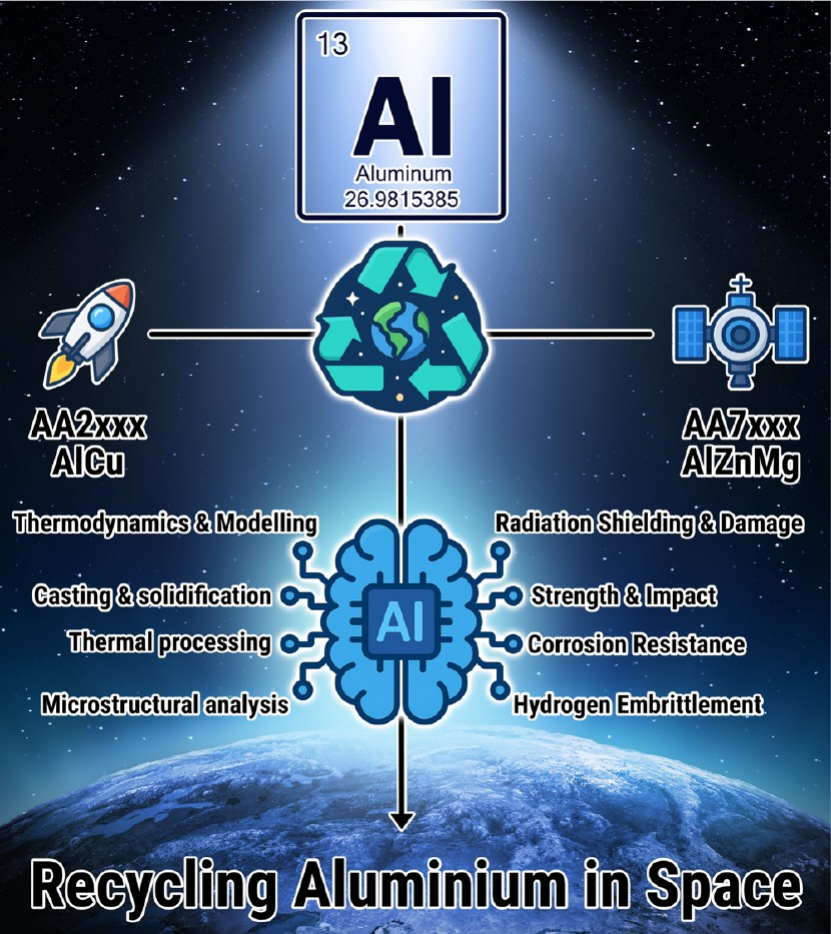
From extraterrestrial origins to AI-driven recyclability in space. In the near future, the combination of supercomputing power and large data sets may enable AI to support metallurgists in recycling space debris composed of diverse aluminum alloys, ultimately creating a unified alloy tailored to meet the requirements of space applications.

## Conclusions

The history of aluminum metallurgy is deeply intertwined with the history of space exploration, and its evolution continues to shape the materials that enable the human race's ambitions beyond Earth. The next generation of aluminum alloys for space applications will emerge from a deliberate fusion of multidisciplinary expertise, integrating advances in alloy design, thermo-mechanical processing, and advanced characterization to counteract the combined effects of radiation and thermal extremes, micrometeoroid impacts, hydrogen embrittlement, and other synergistic degradation forces. By coupling a fundamental understanding of microstructural stability with innovative strategies—such as phase engineering, optimized precipitation pathways, and adaptive manufacturing—it will be possible to develop alloys capable of sustaining exceptional performance over the prolonged lifetimes demanded by deep-space missions. These alloys will safeguard the structural integrity of spacecraft and extraterrestrial habitats, enabling not only the realization of current mission objectives but also expanding the horizons of what is technically achievable in human space exploration. The history of space exploration is, in part, the history of the metallurgy of aluminum evolving to meet the universe’s deepest challenges. One can only anticipate a future of new, exciting discoveries that will support mankind’s journey toward other worlds.

## Footnotes

1 The [X-MAT] team – x-mat.unileoben.ac.at.

2 A.U. = Astronomical Unit.

3 I use this handbook as a reference guide to the composition and uses of soviet aluminum alloys in this paper.

4 Nowadays known as Los Alamos Neutron Scattering Center, LANSCE.

## Notes

I especially dedicate this paper to Professor Peter J. Uggowitzer^1^ on his 75th birthday, with deep gratitude for his lifelong passion and contribution to science, his generous guidance and mentorship of future metallurgists, and his infinite curiosity that reaches from the fundamentals of metallurgy to bold visions like bio and space materials. His example has shaped not only my work but also my life. It is a glorious time to be alive, doing research in metallurgy with you.

## References

[1] Wikipedia contributors, Uggowitzer, P. https://en.wikipedia.org/wiki/Peter_Uggowitzer, 2025 (accessed Sep 17, 2025).

[2] Lewis, J. A. Space exploration in a changing international environment; Rowman & Littlefield: 2014.

[3] Rathnasabapathy M., Slavin M., Wood D. (2024). Role of emerging nations
in ensuring
long-term space sustainability. Acta Astronautica.

[4] López A., Pascuini P., Ramos A. (2018). Climbing the
Space Technology Ladder
in the South: the case of Argentina. Space Policy.

[5] Mkrtchian R., Tkachuk A., Udovenko Z. (2019). Administrative
Legal Support of Outer
Space Activities of the Federative Republic of Brazil. Adv. Space Law.

[6] Gutiérrez, S. D. S. ; Fuentes, C. R. ; Aguilar, A. R. ; de la Rosa Nieves, S. ; Jimenéz, F. J. M. ; Cabrera, E. P. ; Gómez, J. S. ; Pérez, J. A. F. Developing a space program for Mexico. In 2013 6th International Conference on Recent Advances in Space Technologies (RAST); 2013; pp 1053–1057.

[7] Oyewole S. (2017). Space research
and development in Africa. Astropolitics.

[8] Shirah, B. ; Mason, C. E. ; Overbey, E. G. ; Kim, J. ; Pandya, S. ; Gonzalez, Y. ; Persad, A. H. ; Ahmed, M. M. ; Ashemimry, M. N. ; Naseer, M. I. ; Sen, J. In Neuroscience Research in Short-Duration Human Spaceflight; Shirah, B. , Ed.; Academic Press: 2025; pp 1–20.

[9] Fernini, I. ; Al-Naimiy, H. M. Present and Future Space Exploration in the United Arab Emirates. In Proceedings of the International Astronautical Congress; IAC: 2022.

[10] Özalp T. (2009). Space as a
strategic vision for Turkey and its people. Space Policy.

[11] Paikowsky D., Israel I. B. (2009). Science and technology for national development: The
case of Israel’s space program. Acta
Astronaut..

[12] Dougherty, K. A. Australia in Space: A History of a Nation’s Involvement; ATF Press: 2017.

[13] Biddington, B. Space security in the 21st century: roles, responsibilities and opportunities for Australia. Ph.D. thesis, UNSW: Sydney, 2019.

[14] Rattenbury, N. J. Ascending to Space: Critical Perspectives from New Zealand and other Nations; Springer: 2024; pp 273–295.

[15] Nasution H., Diana S. R., Sianipar B., Rubiyanti S., Susanti D., Rafikasari A. (2018). Indonesia Membership on Asia-Pacific
Space Cooperation Organization (APSCO): Cost and Benefit Analysis. Jurnal Hubungan Internasional.

[16] Sivaraks J., Malisuwan S., Kaewphanuekrungsi W. (2021). Space Industry Development: Opportunities
and Challenging in Thailand. International Journal
of Science and Management Studies (IJSMS).

[17] Cottom T. S. (2019). An examination
of Vietnam and space. Space Policy.

[18] Ismail, N. A. Space sector development in Malaysia. In ASEAN Space Programs: History and Way Forward; Springer: 2022; pp 43–55.

[19] Jankowitsch P. (1977). Contributions
of the United Nations Committee on the Peaceful Uses of Outer Space:
An Overview. J. Space Law.

[20] Pyenson L., Mathai A. M., Haubold H. J. (2019). United Nations education program
in space science and technology 1988–2018. Creative Education.

[21] Pelton, J. N. ; Madry, S. Artificial Intelligence for Space: AI4SPACE; CRC Press: 2023; pp 53–104.

[22] Jenkins, M. Routledge Handbook of Space Policy; Routledge: 2025; pp 393–410.

[23] McDougall W. A. (1985). Sputnik,
the space race, and the Cold War. Bulletin of
the atomic scientists.

[24] James C. C. (2023). The cost
of space classification. Ether: J. Strateg.
Airpower Spacepower.

[25] Lehman, M. This High Man: The Life of Robert H. Goddard; Farrar, Straus & Cudahy: New York, 1963; Preface by Charles Lindbergh.

[26] Ashby, M. Materials Selection in Mechanical Design; Elsevier Science: 2017.

[27] Murty, B. S. ; Yeh, J.-W. ; Ranganathan, S. ; Bhattacharjee, P. P. High-entropy alloys; Elsevier: 2019.

[28] Dever, J. ; Banks, B. ; de Groh, K. ; Miller, S. Handbook of environmental degradation of materials; Elsevier: 2005; pp 465–501.

[29] Mouritz, A. P. Introduction to aerospace materials; Elsevier: 2012.

[30] Finckenor, M. M. Chapter Materials for Spacecraft. In Aerospace Materials and Applications; AIAA: 2018; pp 403–434.

[31] Curreri, P. A. Chapter Materials for Exploration Systems. In Aerospace Materials and Applications; AIAA: 2018; pp 505–530.

[32] Ghidini T. (2018). Materials
for space exploration and settlement. Nature
materials.

[33] de
Groh K. K., Banks B. A., Miller S. K., Dever J. A. (2018). Handbook
of Environmental Degradation of Materials. Elsevier.

[34] Tunes M. A., Stemper L., Greaves G., Uggowitzer P. J., Pogatscher S. (2020). Prototypic Lightweight Alloy Design
for Stellar-Radiation
Environments. Adv. Sci..

[35] Willenshofer, P. D. ; Tunes, M. A. ; Vo, H. T. ; Stemper, L. ; Renk, O. ; Greaves, G. ; Uggowitzer, P. J. ; Pogatscher, S. Radiation-resistant aluminium alloy for space missions in the extreme environment of the solar system. 2022; https://arxiv.org/abs/2210.03397. 10.1002/adma.202513450 PMC13054116 41395852

[36] Willenshofer, P. D. Aluminium Alloys in Extreme Environments for Space Applications. PhD thesis, Montanuniversität Leoben: 2024, 10.34901/mul.pub.2025.037.

[37] Hernandez, S. G. Development and Characterisation of an Ultrafine-Grained Al-Mg-Si Alloy for Space Applications. MSc Thesis, Montanuniversität Leoben: 2024; 10.34901/mul.pub.2024.240.

[38] Jones, H. The recent large reduction in space launch cost, 2018.

[39] Wieczerzak K., Nowicka O., Michalski S., Edwards T., Jain M., Xie T., Pethö L., Maeder X., Michler J. (2021). Ultrastrong nanocrystalline
binary alloys discovered via high-throughput screening of the CoCr
system. Materials & Design.

[40] Taylor T. P., Ding M., Ehler D. S., Foreman T. M., Kaszuba J. P., Sauer N. N. (2003). Beryllium in the
environment: a review. Journal of Environmental
Science and Health, Part A.

[41] Willis H. H., Florig H. K. (2002). Potential Exposures and Risks from Beryllium-Containing
Products. Risk Analysis: An International Journal.

[42] Trueman, D. L. ; Sabey, P. Beryllium. In Critical metals handbook; Wiley: 2014; pp 99–121.

[43] Mondolfo, L. F. Aluminum alloys: structure and properties; Elsevier: 2013.

[44] Makar G., Kruger J. (1993). Corrosion of magnesium. International
materials reviews.

[45] Schütze, M. ; Wieser, D. ; Bender, R. Corrosion resistance of aluminium and aluminium alloys; John Wiley & Sons: 2010.

[46] Reboul M., Baroux B. (2011). Metallurgical aspects of corrosion
resistance of aluminium
alloys. Materials and Corrosion.

[47] Mondol S., Alam T., Banerjee R., Kumar S., Chattopadhyay K. (2017). Development
of a high temperature high strength Al alloy by addition of small
amounts of Sc and Mg to 2219 alloy. Materials
Science and Engineering: A.

[48] Rambabu P., Eswara Prasad N., Kutumbarao V., Wanhill R. (2017). Aluminium alloys for
aerospace applications. Aerospace Materials
and Material Technologies: Volume 1: Aerospace Materials.

[49] Nakai M., Eto T. (2000). New aspect of development of high
strength aluminum alloys for aerospace
applications. Materials Science and Engineering:
A.

[50] Li S., Yue X., Li Q., Peng H., Dong B., Liu T., Yang H., Fan J., Shu S., Qiu F., Jiang Q. (2023). Development and applications
of aluminum alloys for aerospace industry. Journal
of Materials Research and Technology.

[51] Sharma A. K. (2005). Surface
engineering for thermal control of spacecraft. Surface engineering.

[52] Abdelal G. F., Abuelfoutouh N., Hamdy A., Atef A. (2007). Thermal fatigue analysis
of small-satellite structure. International
Journal of Mechanics and Materials in Design.

[53] Goueffon Y., Arurault L., Mabru C., Tonon C., Guigue P. (2009). Black anodic
coatings for space applications: study of the process parameters,
characteristics and mechanical properties. Journal
of Materials Processing Technology.

[54] Dunn, B. D. Materials and Processes: for Spacecraft and High Reliability Applications; Springer: 2015; pp 247–328.

[55] Glenny E. (1961). Thermal fatigue. Metall. Rev..

[56] Iyengar, V. K. C. Modelling of the thermal environment and subsystem for a 6U cubesat in GTO orbit. Ph.D. thesis, Luleå University of Technology, Space Technology: 2020.

[57] Juhasz, A. An analysis and procedure for determining space environmental sink temperatures with selected computational results. In Collection of Technical Papers. 35th Intersociety Energy Conversion Engineering Conference and Exhibit (IECEC) (Cat. No.00CH37022); 2000; pp 1175–1183.

[58] Google , Geo Guidelines — Products and Services, Brand Resource Center. https://about.google/brand-resource-center/products-and-services/geo-guidelines/, 2025 (accessed Sep 20, 2025).

[59] Calle, L. Corrosion Control in the Aerospace Industry; Elsevier: 2009; pp 195–224.

[60] Schwighamer, R. A New Era in Space Transportation; Elsevier: 1977; pp 71–88.

[61] Draim, J. ; Stalzer, C. Re-Entry and Vehicle Design; Elsevier: 1960; pp 339–375.

[62] Kirilin A., Shakhmatov E., Soifer V., Akhmetov R., Tkachenko S., Prokofev A., Salmin V., Stratilatov N., Semkin N., Abrashkin V. (2015). Small satellites “AIST”
constellation-design, construction and program of scientific and technological
experiments. Procedia Engineering.

[63] Ramirez O. M. P., Queiroz F. M., Tunes M. A., Antunes R. A., Rodrigues C. L., Lanzutti A., Pogatscher S., Olivier M.-G., De Melo H. G. (2020). Tartaric-sulphuric
acid anodized clad AA2024-T3 post-treated in Ce-containing solutions
at different temperatures: Corrosion behaviour and Ce ions distribution. Appl. Surf. Sci..

[64] NASA . Orion crew module pressure vessel lifted from welding tool. https://commons.wikimedia.org/wiki/File:Orion_crew_module_pressure_vessel_lifted_from_welding_tool_(jsc2022e046165).jpg, 2022; Image courtesy of NASA via Wikimedia Commons.

[65] Byloff J., Trost C. O. W., Devulapalli V., Altaf Husain S., Faurie D., Renault P.-O., Edwards T. E. J., Cordill M. J., Casari D., Putz B. (2025). Atomic Layer-Deposited Interlayers
for Robust Metal–Polymer Interfaces. ACS Appl. Mater. Interfaces.

[66] Prada
Ramirez O., Kremmer T., Marin J., da Silva B., Starykevich M., Tunes M., Ferreira M., Aoki I., Ando R., Pogatscher S., de Melo H. (2023). Ce nanoparticles and
sol-gel hybrid organic-inorganic coatings maximize corrosion protection
in the anodized AA2024-T3. Corros. Sci..

[67] Peltier F., Thierry D. (2022). Review of Cr-free coatings
for the corrosion protection
of aluminum aerospace alloys. Coatings.

[68] Kinard, W. H. ; Martin, G. D. Long duration exposure facility (LDEF) space environments overview. In LDEF: 69 Months in Space. First Post-Retrieval Symposium, Part 1; 1992.

[69] Zook, H. A. Deriving the velocity distribution of meteoroids from the measured meteoroid impact directionality on the various LDEF surfaces. In LDEF: 69 Months in Space. First Post-Retrieval Symposium, Part 1; NASA, Langley Research Center: 1992.

[70] Cour-Palais B. (1999). A career in
applied physics: Apollo through space station. International Journal of Impact Engineering.

[71] Kessler, D. J. Orbital debris environment for spacecraft designed to operate in low Earth orbit; National Aeronautics and Space Administration: 1989; Vol. 100471.

[72] Kessler, D. J. List of Publications. 2025. https://aquarid.physics.uwo.ca/kessler/iwp2.htm (accessed Jul 15, 2025).

[73] NASA . LDEF retrieved by the space shuttle in 1990. 1990. https://commons.wikimedia.org/wiki/File:1990_s32_LDEF_stow.jpg. Image courtesy of NASA via Wikimedia Commons (accessed Jul 15, 2025).

[74] Schwinghamer, R. J. ; Whitaker, A. Shield Design For Protection Against Hypervelocity Particles. https://ntrs.nasa.gov/citations/19930000787, 1993; NASA Marshall Space Flight Center, published in NASA Tech Briefs, Vol. 17, No. 12 (accessed Jul 16, 2025).

[75] Christiansen, E. L. Meteoroid/Debris Shielding. 2003. https://ntrs.nasa.gov/citations/20030068423, NASA Johnson Space Center, Houston (accessed Jul 16, 2025).

[76] Factories in Space/Erik Kulu, In-Space Manufacturing Landscape – April 2024, 2024. https://www.factoriesinspace.com/graphs/Factories-In-Space-ISM-2024-04-01-A3.pdf (accessed Jul 16, 2025).

[77] Frick, J. ; Kulu, E. ; Rodrigue, G. ; Hill, C. ; Senesky, D. G. Semiconductor Manufacturing in Low-Earth Orbit for Terrestrial Use. 2023; osf.io/d6ar4_v1.

[78] Mishima Y., Hori M., Suzuki T., Umekawa S. (1986). Fabrication
of a carbon
fibre/aluminium alloy composite under microgravity. J. Mater. Sci..

[79] Avchare K. R., Tarwani A., Jain D., Saini U., Purohit R. (2014). Space manufacturing
techniques: a review. Int. J. Sci. Res. Publ..

[80] Cesaretti G., Dini E., De Kestelier X., Colla V., Pambaguian L. (2014). Building components
for an outpost on the Lunar soil by means of a novel 3D printing technology. Acta Astronautica.

[81] Prater, T. J. ; Werkheiser, M. J. ; Jehle, A. ; Ledbetter, F. ; Bean, Q. ; Wilkerson, M. ; Soohoo, H. ; Hipp, B. NASA’s in-space manufacturing project: Development of a multimaterial fabrication laboratory for the international space station. In AIAA SPACE and astronautics forum and exposition; AIAA: 2017; p 5277.

[82] Moraguez, M. ; de Weck, O. Benefits of in-space manufacturing technology development for human spaceflight. In 2020 IEEE Aerospace Conference; IEEE: 2020; pp 1–11.

[83] Ishfaq K., Asad M., Mahmood M. A., Abdullah M., Pruncu C. (2022). Opportunities
and challenges in additive manufacturing used in space sector: a comprehensive
review. Rapid Prototyping Journal.

[84] Hoffmann M., Elwany A. (2023). In-space additive manufacturing:
A review. J. Manuf. Sci. Eng..

[85] Makaya A., Pambaguian L., Ghidini T., Rohr T., Lafont U., Meurisse A. (2023). Towards out of earth manufacturing: overview of the
ESA materials and processes activities on manufacturing in space. CEAS Space Journal.

[86] Subeshan B., Ali Z., Asmatulu E. (2024). Metal Additive Manufacturing
in Space and Aerospace
Exploration: Current Processes, Material Selection and Challenges. Journal of Engineering and Applied Sciences.

[87] Subin A. J., Jackson J., Foster J., Silva T., Markham E., Menezes P. L. (2025). In-Space Manufacturing:
Technologies, Challenges, and
Future Horizons. J. Manuf. Mater. Process..

[88] Blakey-Milner B., Gradl P., Snedden G., Brooks M., Pitot J., Lopez E., Leary M., Berto F., Du Plessis A. (2021). Metal additive
manufacturing in aerospace: A review. Materials
& Design.

[89] Tunes M. A., De Oliveira C., Schön C. G. (2017). Multi-objective optimization of a
compact pressurized water nuclear reactor computational model for
biological shielding design using innovative materials. Nuclear engineering and design.

[90] Moldwin, M. An introduction to space weather; Cambridge University Press: 2022.

[91] Office of the Federal Coordinator for Meteorological Services and Supporting Research, National Space Weather Program Strategic Plan. Office of the Federal Coordinator for Meteorological Services and Supporting Research, Technical Report FCM P30-1995, 1995; Technical Report.

[92] Biermann L. (1951). Kometenschweife
und solare Korpuskularstrahlung. Z. Astrophys..

[93] Parker E. N. (1958). Interaction
of the solar wind with the geomagnetic field. Phys. Fluids.

[94] Dessler, A. Solar wind interactions and the magnetosphere. In Physics of the Magnetosphere: Based upon the Proceedings of the Conference Held at Boston College June 19–28, 1967; 1968; pp 65–105.

[95] Mewaldt R. (1994). Galactic cosmic
ray composition and energy spectra. Adv. Space
Res..

[96] Schwadron N. A. (2018). Update on the Worsening Particle Radiation Environment Observed by
CRaTER and Implications for Future Human Deep-Space Exploration. Space Weather.

[97] Baker D. N. (2001). Satellite
anomalies due to space storms. Space storms
and space weather hazards.

[98] Holmes-Siedle, A. ; Adams, L. Handbook of radiation effects; Oxford University Press: 2002.

[99] Schwenn R. (2006). Space weather:
The solar perspective. Liv. Rev. Sol. Phys..

[100] Sznajder M. (2023). Solar wind H+ fluxes at 1 AU for solar cycles 23 and
24. Adv. Space Res..

[101] Ugwumadu C., Drabold D. A., Tutchton R. M. (2025). Effects
of Galactic
Irradiation on Thermal and Electronic Transport in Tungsten. Phys. Status Solidi B.

[102] Blasi P. (2013). The origin of galactic cosmic rays. Astron.
Astrophys. Rev..

[103] Rachman, T. In Physics of the Magnetosphere; Carovillano, R. L. ; McClay, J. F. ; Radoski, H. R. , Eds.; Springer Netherlands: 1968; Vol. 10, pp 10–27.

[104] Mason G. M., Reames D. V., von Rosenvinge T. T., Klecker B., Hovestadt D. (1986). The heavy-ion
compositional signature
in He-3-rich solar particle events. Astrophysical
Journal.

[105] Reames D. V. (1990). Energetic particles from impulsive
solar flares. Astrophysical Journal Supplement
Series.

[106] Reames D. V. (1990). Acceleration of energetic particles
by shock waves
from large solar flares. Astrophysical Journal.

[107] Reames D. V., Meyer J. P., von Rosenvinge T. T. (1994). Energetic-Particle
Abundances in Impulsive Solar Flare Events. International Astronomical Union Colloquium.

[108] Reames D. V. (1995). Coronal abundances determined from
energetic particles. Adv. Space Res..

[109] Nymmik R. A., Panasyuk M. I., Suslov A. A. (1996). Galactic
cosmic
ray flux simulation and prediction. Adv. Space
Res..

[110] Reames D. V. (1996). Energetic
particles from solar flares and coronal mass
ejections. AIP Conf. Proc..

[111] Reames D. V., Ng C. K. (1998). Streaming-limited Intensities of
Solar Energetic Particles. Astrophysical Journal.

[112] Reames D. V. (1999). Particle acceleration at the sun
and in the heliosphere. Space Science Reviews.

[113] Reames D. V. (2000). Abundances of Trans-Iron Elements
in Solar Energetic
Particle Events. Astrophysical Journal.

[114] Kahler S. W., Reames D. V. (2003). Solar Energetic
Particle Production
by Coronal Mass Ejection–driven Shocks in Solar Fast-Wind Regions. Astrophysical Journal.

[115] Christl M., Mangini A., Holzkämper S., Spötl C. (2004). Evidence for
a link between the flux of galactic cosmic
rays and Earth’s climate during the past 200,000 years. Journal of Atmospheric and Solar-Terrestrial Physics.

[116] Reames D. V. (2013). The two sources of solar energetic
particles. Space Science Reviews.

[117] Hassler D. M. (2014). Mars’ surface
radiation environment
measured with the Mars science laboratory’s curiosity rover. Science.

[118] Reames D. V. (2014). Element Abundances in Solar Energetic
Particles and
the Solar Corona. Solar Physics.

[119] Grießmeier J. M., Tabataba-Vakili F., Stadelmann A., Grenfell J. L., Atri D. (2015). Galactic cosmic rays
on extrasolar
Earth-like planets - I. Cosmic ray flux. Astronomy
& Astrophysics.

[120] Morley S. K., Sullivan J. P., Carver M. R., Kippen R. M., Friedel R. H., Reeves G. D., Henderson M. G. (2017). Energetic
Particle Data From the Global Positioning System Constellation. Space Weather.

[121] Wiedenbeck M. E. (2020). 3He-rich Solar Energetic Particle Observations
at the Parker Solar Probe and near Earth. Astrophysical
Journal Supplement Series.

[122] Cohen C. M. (2021). Parker Solar Probe observations of He/H abundance
variations in SEP events inside 0.5 au. Astron.
Astrophys..

[123] Getachew T. (2022). PSP/IS-IS Observation
of a Solar Energetic
Particle Event Associated with a Streamer Blowout Coronal Mass Ejection
during Encounter 6. Astrophysical Journal.

[124] Xu Z. G. (2024). Composition Variation
of the 2023 May 16 Solar Energetic
Particle Event Observed by SolO and PSP. Astrophysical
Journal Letters.

[125] NASA and ESA , SOHO — Solar and Heliospheric Observatory. 2024. https://soho.nascom.nasa.gov/ (accessed Mar 8, 2024).

[126] NASA Science , ACE Mission — Advanced Composition Explorer. 2024. https://science.nasa.gov/mission/ace/ (accessed Mar 8, 2024].

[127] NASA . Wind Mission — NASA.

[128] NASA EOS , Deep Space Climate Observatory (DSCOVR) Mission. 2024. https://eospso.nasa.gov/missions/deep-space-climate-observatory (accessed Mar 8, 2024).

[129] Henk J., Whitelocke R., Warrington A., Bessell E. (1993). Radiation dose to the lens and cataract
formation. Int. J. Radiat. Oncol. Biol. Phys..

[130] Hopewell J. (1990). The skin: its structure and response
to ionizing radiation. International journal
of radiation biology.

[131] Baumüller D., Gehren T. (1997). Aluminium in metal-poor
stars. Astron. Astrophys..

[132] Lodders K. (2003). Solar system abundances and condensation temperatures
of the elements. Astrophysical Journal.

[133] MacPherson G. J. (2003). Calcium-aluminum-rich inclusions
in chondritic meteorites. Treat. Geochem..

[134] Greenwood, N. N. ; Earnshaw, A. Chemistry of the Elements; Elsevier: 2012.

[135] Wikipedia contributors . Hans Christian Ørsted — Wikipedia, the free encyclopedia. 2024. https://en.wikipedia.org/wiki/Hans_Christian_\%C3\%98rsted (accessed Jul 24, 2025).

[136] Ørsted, H. C. Oversigt over det Kongelige Danske Videnskabernes Selskabs Forhandlinger og dets Medlemmers Arbeider, fra 31 Mai 1824 til 31 Mai 1825. In Proceedings of the Royal Danish Academy of Sciences and Letters, Copenhagen; 1825.

[137] Wikipedia contributors . Friedrich Wöhler — Wikipedia, the free encyclopedia, 2024. https://en.wikipedia.org/wiki/Friedrich_W\%C3\%B6hler (accessed Jul 24, 2025).

[138] Wöhler F. (1827). Ueber das aluminium. Annalen
der Physik.

[139] Wikipedia contributors . Henri Étienne Sainte-Claire Deville — Wikipedia, the free encyclopedia, 2024. https://en.wikipedia.org/wiki/Henri_\%C3\%89tienne_Sainte-Claire_Deville (accessed Jul 24, 2025).

[140] Deville, H. É. S.-C. De l’aluminium. Ses propri é t é s, sa fabrication et ses applications; Maleet-Brachelier: 1859.

[141] Lorentsen O.-A. (2014). 125 years of the Hall-Héroult
Process—What
Made It a Success?. Molten salts chemistry and
technology.

[142] Habashi, F. Essential Readings in Light Metals: Vol. 1 Alumina and Bauxite; Springer: 2016; pp 85–93.

[143] Wikipedia contributors. Jules Verne — Wikipedia, the free encyclopedia, 2024. https://en.wikipedia.org/wiki/Jules_Verne (accessed Jul 24, 2025).

[144] Verne, J. From the Earth to the Moon and Around the Moon; Simon and Schuster: 2024.

[145] Goddard, R. H. The Papers of Robert H. Goddard, Volume II: 1925–1937 [1925–1930: Liquid-Propellant Rockets Fly, 1970. https://commons.clarku.edu/cgi/viewcontent.cgi?article=1007&context=papersgoddard, Published by Smithsonian Institution (accessed Jul 24, 2025).

[146] Kablov E., Antipov V., Oglodkova J., Oglodkov M. (2021). Development and application prospects of aluminum–lithium
alloys in aircraft and space technology. Metallurgist.

[147] Charette R. O., Leonard B. G., Bozich W. F., Deamer D. A. (1998). Russian
aluminum-lithium alloys for advanced reusable spacecraft. AIP Conf. Proc..

[148] Rioja R. J., Liu J. (2012). The evolution of Al-Li
base products
for aerospace and space applications. Metallurgical
and Materials Transactions A.

[149] Neufeld, M. J. The rocket and the Reich: Peenem ü nde and the coming of the ballistic missile era; Simon and Schuster: 1995.

[150] Jacobsen, A. Operation Paperclip: The secret intelligence program that brought Nazi scientists to America; Little, Brown: 2014.

[151] Neufeld M. J. (2012). The Nazi aerospace exodus: towards
a global, transnational
history. History and Technology.

[152] Central Intelligence Agency , Handbook of Soviet Alloy Compositions, 1959. https://www.cia.gov/readingroom/document/cia-rdp81-01043r004500060007-5, CREST Report No. CIA-RDP81-01043R004500060007-5; 133 pages; originally classified “K”; published 25 August 1959; released 20 May 2014.

[153] WikiImages . Sputnik Satellite, 2015. https://pixabay.com/photos/sputnik-satellite-astronautics-nasa-986/, Image courtesy of WikiImages via Pixabay, Pixabay License (accessed Jul 24, 2025).

[154] Pline, Vostok 1 capsule at the Musée de l’Air et de l’Espace, Le Bourget, 2005. https://commons.wikimedia.org/wiki/File:Vostok-1-musee-du-Bourget-P.jpg, Image licensed under CC BY-SA 2.5 via Wikimedia Commons (accessed Jul 24, 2025).

[155] Duyunova V. A., Leonov A. A., Molodtsov S. V. (2020). The Contribution
of VIAM to the Development of Lightweight Alloys and Corrosion Protection
of Rocket and Space Technology Products (in Russian). Proceedings of VIAM.

[156] Editorial, Iosif Naumovich Fridlyander (on his 95th birthday). Russian Metallurgy (Metally) 2008, 2008 447–448. 10.1134/S0036029508050145

[157] Glynn, P. C. ; Moser, T. L. Orbiter structural design and verification. In NASA Johnson Space Center Space Shuttle Tech. Conf., Pt. 1; 1985; pp 345–356.

[158] Schneider, W. C. ; Miller, G. J. The Challenging of Scales of the Bird (Shuttle Tile Structural Integrity). In NASA Johnson Space Center Space Shuttle Tech. Conf., Pt. 1; 1985; pp 403–413.

[159] Williams, J. ; Oren, J. ; Modest, M. ; Howell, H. Challenges in the development of the orbiter radiator system. In NASA Johnson Space Center Space Shuttle Tech. Conf., Pt. 1; 1985; pp 480–489.

[160] Vogl G., Weiss B. (1965). Der Einfluss von neutronenbestrahlung
auf die ausscheidungskinetik iv einer übersättigten
Al-Cu-Legierung. Acta Metall..

[161] Katz L., Herman H., Damask A. (1968). Precipitation
in neutron-irradiated
Al-base Cu. Acta Metall..

[162] Liu K., Kawano O., Murakami Y., Yoshida H. (1972). Structural changes
in age-hardenable aluminium alloys induced by low temperature neutron
irradiation. Radiation Effects.

[163] Farrell K. (1981). Microstructure and tensile properties
of heavily irradiated
5052–0 aluminum alloy. J. Nucl. Mater..

[164] Piatti G., Fiorini P., Schiller P. (1984). High purity
aluminium
alloys for experimental fusion reactors. Nuclear
Engineering and Design. Fusion.

[165] Böning K., Von Der Hardt P. (1987). Physics and
safety of advanced research
reactors. Nuclear Instruments and Methods in
Physics Research Section A: Accelerators, Spectrometers, Detectors
and Associated Equipment.

[166] Ismail Z. (1990). Effect of low dose neutron irradiation on the mechanical
properties of an AIMgSi alloy. Radiat. Eff.
Defects Solids.

[167] Ghauri I., Afzal N. (2007). Effects of neutron irradiation on
the stress relaxation rate in Al–Cu–Mg alloy. J. Phys. D: Appl. Phys..

[168] Kolluri, M. Neutron irradiation effects in 5xxx and 6xxx series aluminum alloys: A literature review. In Radiat. Eff. Mater.; IntechOpen: 2016; Vol. 10, p 63294.

[169] Tunes M. A., Greaves G., Kremmer T. M., Vishnyakov V. M., Edmondson P. D., Donnelly S. E., Pogatscher S., Schön C. G. (2019). Thermodynamics of an austenitic stainless steel (AISI-348)
under in situ TEM heavy ion irradiation. Acta
Mater..

[170] Lohmann W., Ribbens A., Sommer W., Singh B. (1987). Microstructure
and mechanical properties of medium energy (600–800 MeV) proton
irradiated commercial aluminium alloys. Radiation
effects.

[171] Andersen S., Zandbergen H., Jansen J., Traeholt C., Tundal U., Reiso O. (1998). The crystal structure of the β″
phase in Al–Mg–Si alloys. Acta
Mater..

[172] Buchanan K., Colas K., Ribis J., Lopez A., Garnier J. (2017). Analysis of
the metastable precipitates in peak-hardness
aged Al-Mg-Si (-Cu) alloys with differing Si contents. Acta Mater..

[173] Pogatscher S., Antrekowitsch H., Leitner H., Ebner T., Uggowitzer P. J. (2011). Mechanisms controlling the artificial aging of Al–Mg–Si
Alloys. Acta Mater..

[174] Flament C., Ribis J., Garnier J., Serruys Y., Leprêtre F., Gentils A., Baumier C., Descoins M., Mangelinck D., Lopez A., Colas K., Buchanan K., Donnadieu P., Deschamps A. (2017). Stability
of β″ nano-phases
in Al-Mg-Si (-Cu) alloy under high dose ion irradiation. Acta Mater..

[175] Hirsch P., Horne R., Whelan M. (1956). LXVIII. Direct observations
of the arrangement and motion of dislocations in aluminium. Philos. Mag..

[176] Mazey D., Bullough R., Brailsford A. (1976). Observation
and analysis of damage structure in Al and Al/Mg (N4) alloy after
irradiation with 100 and 400 keV aluminium ions. J. Nucl. Mater..

[177] Hirsch P. (1986). Direct observations of moving dislocations: reflections
on the thirtieth anniversary of the first recorded observations of
moving dislocations by transmission electron microscopy. Materials Science and Engineering.

[178] Stemper L., Tunes M. A., Oberhauser P., Uggowitzer P. J., Pogatscher S. (2020). Age-hardening response of AlMgZn
alloys with Cu and Ag additions. Acta Mater..

[179] Stemper L., Tunes M. A., Dumitraschkewitz P., Mendez-Martin F., Tosone R., Marchand D., Curtin W. A., Uggowitzer P. J., Pogatscher S. (2021). Giant hardening response in AlMgZn
(Cu) alloys. Acta Mater..

[180] Stemper L., Tunes M. A., Tosone R., Uggowitzer P. J., Pogatscher S. (2022). On the potential of aluminum crossover
alloys. Prog. Mater. Sci..

[181] Schmid, F. ; Stemper, L. ; Tosone, R. AMAG crossalloy—a unique aluminum alloy concept for lightweighting the future. In TMS Annual Meeting & Exhibition; 2023; pp 500–504.

[182] Stemper, L. ; Schmid, F. ; Tosone, R. AMAG crossalloy®—lightweighting the future by unconstraint alloy design: A case study. In TMS Annual Meeting & Exhibition; 2024; pp 241–247.

[183] Ohba T., Kitano Y., Komura Y. (1984). The charge-density
study of the Laves phases, MgZn2 and MgCu2. Crystal Structure Communications.

[184] Bergman G., Waugh J. L., Pauling L. (1957). The crystal structure
of the metallic phase Mg32 (Al, Zn) 49. Acta
Crystallogr..

[185] Owen E., Preston G. (1923). The atomic structure of two intermetallic
compounds. Proceedings of the Physical Society
of London.

[186] Granberg F., Nordlund K., Ullah M. W., Jin K., Lu C., Bei H., Wang L., Djurabekova F., Weber W., Zhang Y. (2016). Mechanism of radiation damage reduction
in equiatomic multicomponent single phase alloys. Physical review letters.

[187] Tunes M. A., Greaves G., Bei H., Edmondson P. D., Zhang Y., Donnelly S. E., Schön C. G. (2021). Comparative
irradiation response of an austenitic stainless steel with its high-entropy
alloy counterpart. Intermetallics.

[188] Velişa G., Granberg F., Levo E., Zhou Y., Fan Z., Bei H., Tuomisto F., Nordlund K., Djurabekova F., Weber W., Zhang Y. (2023). Recent progress
on understanding
the temperature-dependent irradiation resistance ranking among NiFe,
NiCoCr, and NiCoFeCr alloys: A review. J. Mater.
Res..

[189] Prangnell P., Bowen J. R., Apps P. (2004). Ultra-fine grain structures
in aluminium alloys by severe deformation processing. Materials Science and Engineering: A.

[190] Valiev R. Z., Langdon T. G. (2006). Principles of equal-channel
angular
pressing as a processing tool for grain refinement. Prog. Mater. Sci..

[191] Langdon T. G. (2007). The processing of ultrafine-grained materials through
the application of severe plastic deformation. J. Mater. Sci..

[192] Estrin, Y. ; Murashkin, M. ; Valiev, R. Fundamentals of aluminium metallurgy; Elsevier: 2011; pp 468–503.

[193] Qiao X. G., Gao N., Moktadir Z., Kraft M., Starink M. J. (2010). Fabrication of MEMS components using ultrafine-grained
aluminium alloys. Journal of Micromechanics
and Microengineering.

[194] He T., Chen S., Lu T., Zhao P., Chen W., Scudino S. (2020). High-strength and ductile ultrafine-grained
Al–Y–Ni–Co
alloy for high-temperature applications. J.
Alloys Compd..

[195] Harsha S., Dasharath S. (2021). Effect of cryogenic heat treatment
& ageing on ultra fine grained aluminium–lithium alloy-A
review. Materials Today: Proceedings.

[196] Orłowska M., Olejnik L., Campanella D., Buffa G., Morawiński Ł., Fratini L., Lewandowska M. (2020). Application
of linear friction welding for joining ultrafine grained aluminium. Journal of Manufacturing Processes.

[197] Langdon T. G. (2013). Twenty-five years of ultrafine-grained
materials: Achieving
exceptional properties through grain refinement. Acta Mater..

[198] Huang Y., Langdon T. G. (2013). Advances in ultrafine-grained materials. Mater. Today.

[199] Chinh N. Q., Murashkin M. Y., Bobruk E. V., Lábár J. L., Gubicza J., Kovács Z., Ahmed A. Q., Maier-Kiener V., Valiev R. Z. (2021). Ultralow-temperature superplasticity and its novel
mechanism in ultrafine-grained Al alloys. Materials
Research Letters.

[200] Zhang X., Hattar K., Chen Y., Shao L., Li J., Sun C., Yu K., Li N., Taheri M. L., Wang H., Wang J., Nastasi M. (2018). Radiation damage in
nanostructured materials. Prog. Mater. Sci..

[201] El-Atwani O., Esquivel E., Efe M., Aydogan E., Wang Y., Martinez E., Maloy S. A. (2018). Loop and
void damage
during heavy ion irradiation on nanocrystalline and coarse grained
tungsten: Microstructure, effect of dpa rate, temperature, and grain
size. Acta Mater..

[202] Nahavandian M., Aydogan E., Byggmästar J., Tunes M. A., El-Atwani O., Martinez E. (2025). Design kinetic parameters
for improved resilience of materials under irradiation. Materials & Design.

[203] Willenshofer P., Tunes M. A., Kainz C., Renk O., Kremmer T. M., Gneiger S., Uggowitzer P. J., Pogatscher S. (2023). Precipitation behaviour in AlMgZnCuAg crossover alloy
with coarse and ultrafine grains. Materials
Research Letters.

[204] Willenshofer P., Coradini D. S. R., Renk O., Uggowitzer P., Tunes M. A., Pogatscher S. (2024). Comparative analysis of experimental
techniques for microstructural characterization of novel nanostructured
aluminium alloys. Mater. Charact..

[205] Theissing, M. ; Gonzaga, S. ; Willenshofer, P. ; Kremmer, T. M. ; Mitsche, S. ; Pogatscher, S. ; Weißensteiner, I. ; Tunes, M. A. Thermal stability and recrystallisation of ultrafine-grained aluminium alloys studied with *in situ* EBSD and TEM. Unpublished manuscript, 2025; Manuscript submitted and under review.

[206] Wu S., Soreide H. S., Chen B., Bian J., Yang C., Li C., Zhang P., Cheng P., Zhang J., Peng Y., Liu G., Li Y., Roven H. J., Sun J. (2022). Freezing solute atoms
in nanograined aluminum alloys via high-density vacancies. Nat. Commun..

[207] Chadwick M. (2006). ENDF/B-VII.0: Next Generation
Evaluated Nuclear
Data Library for Nuclear Science and Technology. Nuclear Data Sheets.

[208] International Atomic Energy Agency . Evaluated Nuclear Data File (ENDF), 2025. https://www-nds.iaea.org/exfor/endf.htm (accessed Aug 11, 2025).

[209] Chao Z., Jiang L., Chen G., Qiao J., Z Q., Yu Z., Cao Y., Wu G. (2019). The microstructure
and ballistic performance of B4C/AA2024 functionally graded composites
with wide range B4C volume fraction. Composites
Part B: Engineering.

[210] Senthilvelan T., Gopalakannan S., Vishnuvarthan S., Keerthivaran K. (2013). Fabrication and characterization
of SiC, Al2O3 and
B4C reinforced Al-Zn-Mg-Cu alloy (AA 7075) metal matrix composites:
a study. Adv. Mater. Res..

[211] Canakci A., Arslan F., Varol T. (2013). Effect of
volume fraction
and size of B4C particles on production and microstructure properties
of B4C reinforced aluminium alloy composites. Mater. Sci. Technol..

[212] Zheng R., Hao X., Yuan Y., Wang Z., Ameyama K., Ma C. (2013). Effect of high volume
fraction of
B4C particles on the microstructure and mechanical properties of aluminum
alloy based composites. Journal of alloys and
compounds.

[213] Wu C., Ma K., Zhang D., Wu J., Xiong S., Luo G., Zhang J., Chen F., Shen Q., Zhang L., Lavernia E. J. (2017). Precipitation phenomena in Al-Zn-Mg alloy matrix composites
reinforced with B4C particles. Sci. Rep..

[214] Shetty R. P., Raju T. H., Nagaral M., Kumar N., Auradi V. (2024). Effect of B4C particles addition on the mechanical,
tensile fracture and wear behavior of Al7075 alloy composites. J. Bio- Tribo-Corros..

[215] Silva F., Prada Ramirez O., Manso A., Österreicher J., Rossino L., Almeida L., Junior C., Sagás J., Consani D., Marzo F., Tunes M. (2025). Is nitrogen doping
of diamond-like carbon films a viable strategy for bipolar plates
in proton exchange membrane fuel cells?. Vacuum.

[216] National Aeronautics and Space Administration. Radiation Hardness Assurance for Space Systems, 1996. https://extapps.ksc.nasa.gov/Reliability/Documents/Preferred_Practices/1258.pdf, Preferred Reliability Practice No. PT-TE-1258, NASA Kennedy Space Center.

[217] Messenger, G. C. ; Ash, M. S. The effects of radiation on electronic systems; Springer: 1986.

[218] Ziegler J.
F., Ziegler M. D., Biersack J. P. (2010). SRIM–The
stopping and range of ions in matter (2010). Nuclear Instruments and Methods in Physics Research Section B: Beam
Interactions with Materials and Atoms.

[219] Stoller R. E., Toloczko M. B., Was G. S., Certain A. G., Dwaraknath S., Garner F. A. (2013). On the use of SRIM
for computing
radiation damage exposure. Nuclear instruments
and methods in physics research section B: beam interactions with
materials and atoms.

[220] Weber W. J., Zhang Y. (2019). Predicting damage production in monoatomic
and multi-elemental targets using stopping and range of ions in matter
code: Challenges and recommendations. Curr.
Opin. Solid State Mater. Sci..

[221] Azadegan N., Hassanpour M., Khandaker M. U., Faruque M. R. I., Al-Mugren K., Bradley D. (2021). Calculation of secondary
radiation absorbed doses due to the proton therapy on breast cancer
using MCNPX code. Radiat. Phys. Chem..

[222] Bernabeu J., Casanova I. (2007). Geant4-based radiation
hazard assessment
for human exploration missions. Adv. Space Res..

[223] Johnson W. H. (1875). On some remarkable
changes produced
in iron and steel by the action of hydrogen and acids. Proc. R. Soc. London.

[224] Johnson W. H. (1875). On some remarkable changes produced
in iron and steel
by the action of hydrogen and acids. Nature.

[225] Djukic M. B., Bakic G. M., Zeravcic V. S., Sedmak A., Rajicic B. (2016). Hydrogen embrittlement of industrial
components: prediction,
prevention, and models. Corrosion.

[226] Djukic M. B., Bakic G. M., Zeravcic V. S., Sedmak A., Rajicic B. (2019). The synergistic action and interplay
of hydrogen embrittlement
mechanisms in steels and iron: Localized plasticity and decohesion. Eng. Fract. Mech..

[227] Tunes M. A., Uggowitzer P. J., Dumitraschkewitz P., Willenshofer P., Samberger S., da Silva F. C., Schön C. G., Kremmer T. M., Antrekowitsch H., Djukic M. B., Pogatscher S. (2024). Limitations
of hydrogen detection after 150 years of research on hydrogen embrittlement. Adv. Eng. Mater..

[228] Zhao H., Chakraborty P., Ponge D., Hickel T., Sun B., Wu C.-H., Gault B., Raabe D. (2022). Hydrogen trapping and
embrittlement in high-strength Al alloys. Nature.

[229] Jiang S. (2025). Structurally complex
phase engineering enables hydrogen-tolerant
Al alloys. Nature.

[230] Yu H., Díaz A., Lu X., Sun B., Ding Y., Koyama M., He J., Zhou X., Oudriss A., Feaugas X., Zhang Z. (2024). Hydrogen embrittlement
as a conspicuous
material challenge: comprehensive review and future directions. Chem. Rev..

[231] Fruchart D., Skryabina N., de Rango P., Fouladvind M., Aptukov V. (2023). Severe plastic deformation by fast forging to easy
produce hydride from bulk Mg-based alloys. Materials
Transactions.

[232] Gray
III G. (1988). Deformation twinning in Al-4.8 wt% Mg. Acta
Metall..

[233] Falco R., Bagherifard S. (2025). Cold spray additive manufacturing:
A review of shape control challenges and solutions. J. Therm. Spray Technol..

[234] Aster P., Dumitraschkewitz P., Uggowitzer P. J., Weißensteiner I., Tunes M. A., Schmid F., Stemper L., Pogatscher S. (2025). Effect of long-term aging and Cu addition on clustering,
strength and strain hardening in Al-Mg-Zn-(Cu) crossover alloys. Materials & Design.

[235] Balazs A. C. (2007). Modeling self-healing materials. Mater. Today.

[236] Wool R. P. (2008). Self-healing
materials: a review. Soft Matter.

[237] Hager M. D., Greil P., Leyens C., Zwaag S. V. D., Schubert U. S. (2010). Self-Healing Materials. Adv.
Mater..

[238] Yang Y., Ding X., Urban M. W. (2015). Chemical
and physical
aspects of self-healing materials. Prog. Polym.
Sci..

[239] Roppolo I., Caprioli M., Pirri C. F., Magdassi S. (2024). 3D Printing
of Self-Healing Materials. Adv. Mater..

[240] Pernigoni L., Lafont U., Grande A. M. (2021). Self-healing materials
for space applications: overview of present development and major
limitations. CEAS Space Journal.

[241] Pernigoni L., Lafont U., Grande A. M. (2024). Assessment
of radiation
shielding properties of self-healing polymers and nanocomposites for
a space habitat case study under GCR and LEO radiation. CEAS Space Journal.

[242] Ackland G. (2010). Controlling radiation damage. Science.

[243] Lumley, R. Self Healing Materials: An Alternative Approach to 20 Centuries of Materials Science; Springer: 2007; pp 219–254.

[244] van Dijk N., van der Zwaag S. (2018). Self-healing phenomena in metals. Adv. Mater. Interfaces.

[245] Ludwig A., Mogeritsch J., Kolbe M., Zimmermann G., Sturz L., Bergeon N., Billia B., Faivre G., Akamatsu S., Bottin-Rousseau S., Voss D. (2012). Advanced Solidification
Studies on Transparent Alloy Systems: A New European Solidification
Insert for Material Science Glovebox on Board the International Space
Station. JOM.

[246] Ludwig A., Mogeritsch J. P. (2023). Observation
of peritectic couple
growth for a hyper-peritectic alloy under microgravity conditions. IOP Conference Series: Materials Science and Engineering.

[247] Abolhasani M., Brown K. A., Guest
Editors (2023). Role of AI in experimental
materials
science. MRS Bull..

[248] Nazarahari A., Fromm A., Ozdemir H., Klose C., Maier H., Canadinc D. (2023). Determination of thermal conductivity
of eutectic Al–Cu compounds utilizing experiments, molecular
dynamics simulations and machine learning. Modell.
Simul. Mater. Sci. Eng..

[249] Ozdemir H., Canadinc D., El Atwani O., Valdez J., Lovato B., Mathews C., Wanni J., Cooley J., Fensin S. (2025). Utilizing machine learning to predict
tensile ductility and yield strength of CoNiV-based multi-principal
elements alloys. Materials & Design.

[250] Canadinc D., Breitbach E., Catal A. (2025). Characterization of
Two Novel NiTiHf Shape Memory Alloys Designed by Machine Learning
Utilizing Novel Experimental Techniques. Shape
Mem. Superelast..

[251] Raabe D., Ponge D., Uggowitzer P. J., Roscher M., Paolantonio M., Liu C., Antrekowitsch H., Kozeschnik E., Seidmann D., Gault B. (2022). Making
sustainable aluminum by recycling scrap: The science of “dirty”
alloys. Prog. Mater. Sci..

